# Antimicrobial d-amino acid oxidase-derived peptides specify gut microbiota

**DOI:** 10.1007/s00018-020-03755-w

**Published:** 2021-01-23

**Authors:** Giulia Murtas, Silvia Sacchi, Gabriella Tedeschi, Elisa Maffioli, Eugenio Notomista, Valeria Cafaro, Monica Abbondi, Jean-Pierre Mothet, Loredano Pollegioni

**Affiliations:** 1 Department of Biotechnology and Life Sciences, University of Insubria, Via J. H. Dunant 3, 21100 Varese, Italy; 2 DAAIR, D-Amino Acid International Research Center, Gerenzano, Italy; 3 Department of Veterinary Medicine, University of Milan, Milan, Italy; 4 Cimaina, University of Milan, Milan, Italy; 5 Department of Biology, University of Naples Federico II, Naples, Italy; 6 Fondazione Istituto Insubrico Ricerca Per La Vita (FIIRV), Gerenzano, Italy; 7 LuMIn, Université Paris-Saclay, CNRS, ENS Paris-Saclay, CentraleSupélec, 91190 Gif-sur-Yvette, France

**Keywords:** d-Amino acids, Microbiota selection, Novel function, Flavoprotein

## Abstract

The flavoenzyme d-amino acid oxidase (DAAO) is deputed to the degradation of d-enantiomers of amino acids. DAAO plays various relevant physiological roles in different organisms and tissues. Thus, it has been recently suggested that the goblet cells of the mucosal epithelia secrete into the lumen of intestine, a processed and active form of DAAO that uses the intestinal d-amino acids to generate hydrogen peroxide (H_2_O_2_), an immune messenger that helps fighting gut pathogens, and by doing so controls the homeostasis of gut microbiota. Here, we show that the DAAO form lacking the 1–16 amino acid residues (the putative secretion signal) is unstable and inactive, and that DAAO is present in the epithelial layer and the mucosa of mouse gut, where it is largely proteolyzed. In silico predicted DAAO-derived antimicrobial peptides show activity against various Gram-positive and Gram-negative bacteria but not on Lactobacilli species, which represent the commensal microbiota. Peptidomic analysis reveals the presence of such peptides in the mucosal fraction. Collectively, we identify a novel mechanism for gut microbiota selection implying DAAO-derived antimicrobial peptides which are generated by intestinal proteases and that are secreted in the gut lumen. In conclusion, we herein report an additional, ancillary role for mammalian DAAO, unrelated to its enzymatic activity.

**Supplementary Information:**

The online version contains supplementary material available at 10.1007/s00018-020-03755-w.

## Introduction

d-amino acids are natural biomolecules, components of human diet, which play specific roles; for a review, see Ref. [1]. d-amino acids are constituents of the peptidoglycan in the bacterial cell wall: their presence makes the cell wall more resistant to proteases and to some antibiotics [2]. Peptidoglycan is a dynamic structure which is modified by introducing alternative d-amino acids during stationary phase by specific periplasmic enzymatic activities [3, 4]: this represents a mechanism of bacteria selection.

The FAD-dependent enzyme d-amino acid oxidase (DAAO, EC 1.4.3.3) catalyzes the oxidative deamination of uncharged and basic d-amino acids [5, 6]. In mammals, DAAO is present in different organs and is expressed by many cell types. In the brain, DAAO might influence the physiology of neuronal circuits by metabolizing d-serine (d-Ser), an allosteric neuromodulator of the *N*-methyl-d-aspartate type of glutamate receptors [7] and therefore is suspected to play a critical role in several psychiatric and neurological disorders [8, 9]. However, the highest expression and activity of DAAO are more apparent in peripheral organs and notably in the proximal tubules of the kidney, in hepatocytes, in neutrophils, and in the goblet cells and enterocytes of the small intestine [6, 10]. In liver and kidney, DAAO is responsible for the detoxification and degradation of d-amino acids originating from the cell wall of intestinal bacteria, diet, and endogenous racemization. In neutrophils and intestinal mucosa, DAAO shows an antimicrobial activity [11, 12]. In the intestine, DAAO is detected in the goblet cells and enterocytes of the villus epithelium [11] of the proximal and middle small intestine of mice and humans. According to a recent report, goblet cells secrete a processed and active form of mouse DAAO (mDAAO), likely due to the presence of a signal peptide and a predicted cleavage site near the *N* terminus [11]. Then, the secreted DAAO uses intestinal d-amino acids originating from the host or from the commensal bacteria to generate bactericidal H_2_O_2_ to help limiting the colonization of enteropathogens including *Staphylococcus aureus* and *Vibrio cholera*.

Intriguingly, structural details support that DAAO cannot properly fold and maintain enzymatic activity (i.e., H_2_O_2_ production) in the absence its *N*-terminal sequence which is part of the Rossmann fold, i.e., the motif required for FAD binding. Therefore, in this work, we employed biochemical, microbial, cellular, and tissue analyses to shed light on the role of the flavoenzyme in antibacterial activity and gut homeostasis. We demonstrate that the bactericide and microbiota selective abilities of DAAO are mainly due to the generation in the gut of specific antimicrobial peptides and not by the generation of H_2_O_2_, as previously proposed. This investigation identifies an additional, ancillary role for mammalian DAAO, unrelated to its enzymatic activity.

## Results

### Sequence analyses of DAAO

**Fig. 1:**
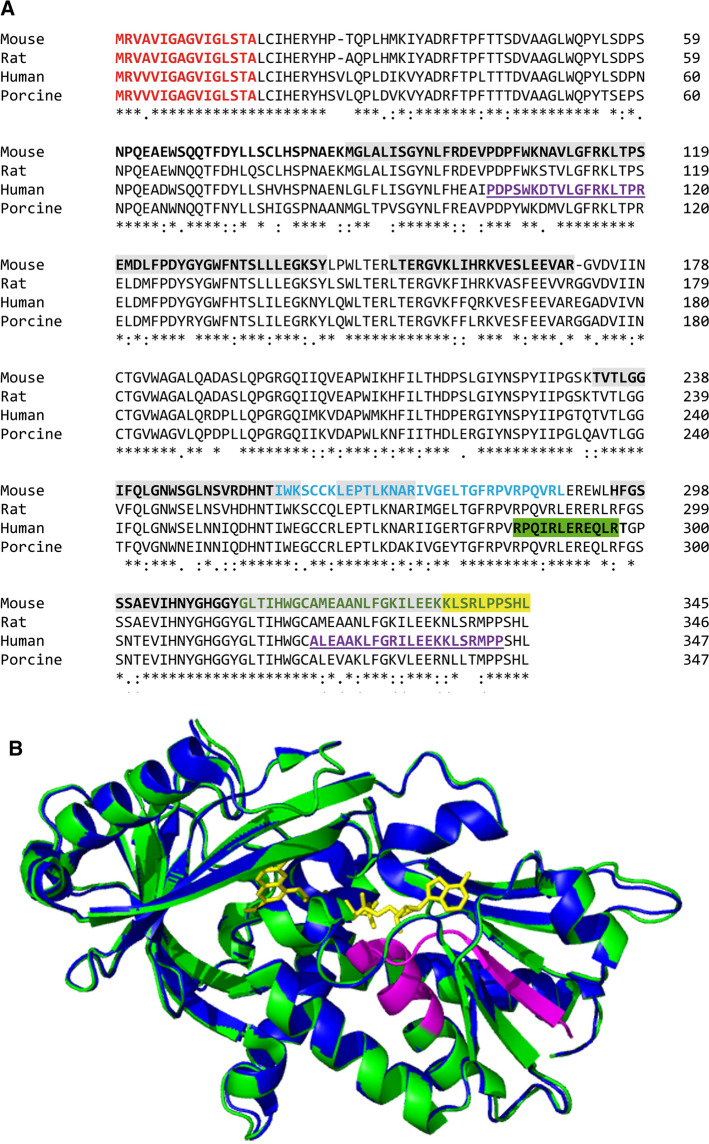
**a** Comparison of the primary structure of mouse, rat, porcine, and human DAAO. The putative secretion signal is marked in red, the regions recognized by anti-*N*-terminal and anti-*C*-terminal hDAAO antibodies are in purple and underlined, and putative antimicrobial sequences are in cyan and green. The regions recognized by the anti-DAAO antibodies used in Ref. [11] are highlighted in green and yellow. The peptides identified by MS analysis in the intestine samples following SDS-PAGE are highlighted in gray. **b** Comparison of the structure of full-length hDAAO (PDB 2DU8, green) and of the model of hDAAO (Δ1–16) variant (blue). The deleted *N*-terminal sequence is colored in magenta

To reveal the presence of a signal peptide and a cleavage site for protein secretion, the sequences of mammalian DAAOs (human, mouse, rat, and porcine) were analyzed in silico with various bioinformatics programs, including the SignalP 3.0 used by Sasabe et al. [11], see Supplemental Data 1. While the SignalP 4.1 Server predicted a signal sequence and a cleavage site between positions 16 and 17 at the *N* terminus of the pig DAAO only (score value of 0.455 vs. a cut-off value of 0.450), three alternative bioinformatics tools predicted the same signal peptide for all the mammalian DAAOs analyzed, while the Signal-3L predicted the presence of this signal peptide in mouse and rat DAAOs only (Fig. 1a and Supplementary Table 1). The human DAAO, hDAAO, (Δ1–16) variant lacking the *N*-terminal 16 residues was thus designed based on these analyses and a previous study stating that the 16 first residues correspond to a secretion signal that was removed in secreted DAAO [11].

The prediction analysis for cryptic antimicrobial peptides (CAMP) in the DAAO sequence suggests the presence of two cryptic cationic peptides, Fig. 1a. The antimicrobial absolute score, AS value which depends on hydrophobicity, net charge, and length of a peptide [13], was calculated for all possible peptides of 12–40 residues length. The highest AS was found for a 33-residues long peptide starting at position 257 (^257^IWKSCCKLEPTLKNARIVGELTGFRPVRPQVRL^289^, named IWK, corresponding to ^259^IWEGCCKLEPTLKNARIIGERTGFRPVRPQIRL^291^ in the human enzyme): the AS value for this peptide is higher than two of the three threshold scores, Supplementary Fig. 1. At the *C* terminus of DAAO, an additional region showed two relative maxima with AS = 4.5 (for the 22 residue window) and AS = 3.3 (for the 33 residue window) corresponding to peptides ^324^AANLFGKILEEKKLSRLPPSHL^345^ and ^313^GLTIHWGCAMEAANLFGKILEEKKLSRLPPSHL^345^, respectively. Even if the score of these peptides is below the threshold, we selected peptide 313–345 (named GLT and corresponding to ^315^GLTIHWGCALEAAKLFGRILEEKKLSRMPPSHL^347^ in the human enzyme) for further characterization, because in the crystal structure of DAAO, this region is structured as a long and amphipathic *α*-helix, a feature often associated with CAMPs. Notably, the T256–I257 and L289–E290 peptide bonds are both recognized by pepsin (cleavages that generate the IWK peptide), and the Y311–G312 bond is a cleavage site for both chymotrypsin and pepsin (and generates the GLT peptide) as suggested by PeptideCutter at ExPASy website.

### The hDAAO (Δ1–16) variant is unstable and inactive

At first, hDAAO (Δ1–16) was produced in *E. coli* BL21DE3Star cells using the conditions reported for the wild-type enzyme [14]: the resulting expression level in the crude extract (< 1 mg/L fermentation broth) was too low for a biochemical characterization. To increase the expression yield of the soluble hDAAO variant, a number of experimental parameters were evaluated as well as the two-step protocol for the inhibition of protein synthesis [15]. Best conditions are reported in Supplementary Data 2 and Supplementary Table 2. When the hDAAO (Δ1–16) variant was purified employing the procedure used for the wild-type enzyme [14], about 50% of the total recombinant hDAAO variant was lost in the ammonium sulfate precipitation step and part (~ 25%) was eluted in the flow through of the DEAE Sepharose FF column: the resulting overall purification yield was extremely low corresponding to 0.04 mg of pure hDAAO variant/L fermentation broth. Indeed, no DAAO activity was detected in the crude extract or in the final enzyme preparation even in the presence of a saturating FAD dose, i.e., 0.2 mM.

To facilitate the production of the deleted variant, and since hDAAO is a stable homodimer in solution [14], it was co-expressed with the wild-type hDAAO using the pET-Duet plasmid as tagged proteins, i.e., His-hDAAO wild-type and strep-hDAAO (Δ1–16). No DAAO enzymatic activity was detected in the crude extract of BL21(DE3)pLysS *E. coli* cells harboring the pET-Duet vector. Neither a HiTrap chelating chromatography nor a StrepTrap column allowed binding of the recombinant proteins. None of the conditions reported in Supplementary Table 3 allowed to produce a suitable amount of hDAAO (Δ1–16).

**Fig. 2:**
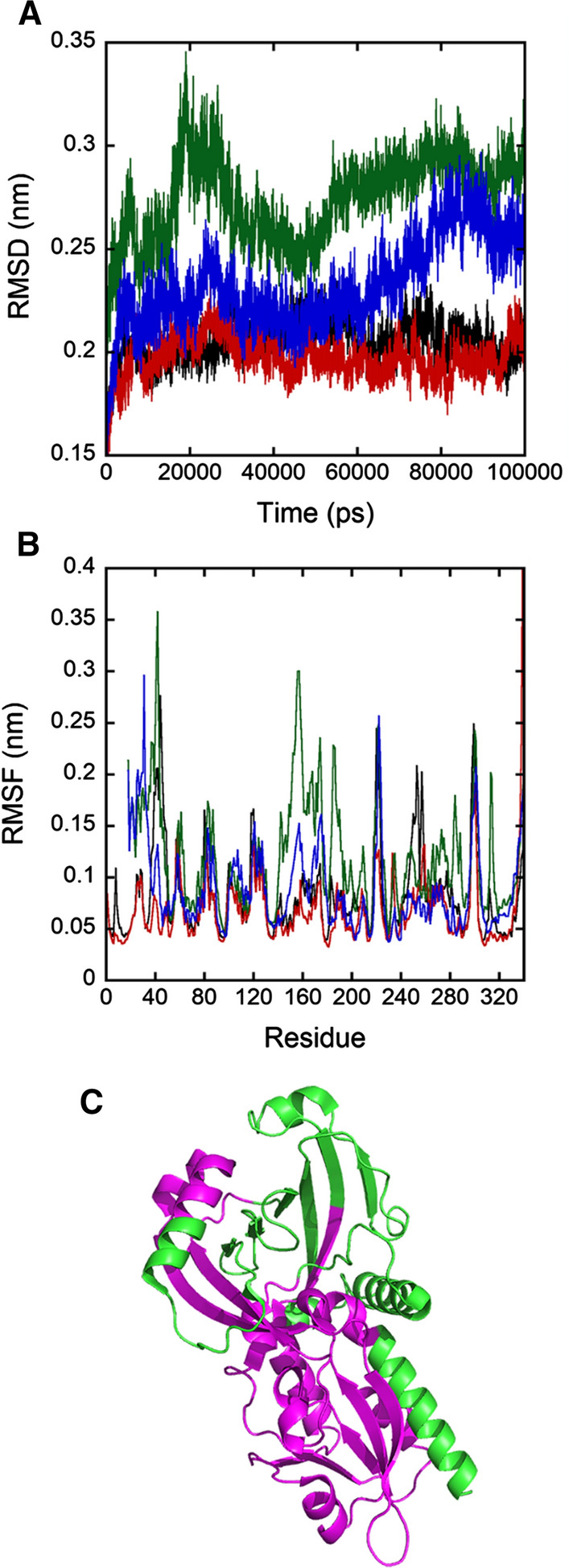
**a** Protein RMSD from the starting structure is represented as a function of simulation time: full-length hDAAO in absence and in presence of FAD (in black and red) and hDAAO (Δ1–16) variant in absence and in presence of the cofactor (in green and blue). **b** The per residue RMSF values are represented as a function of the residue number (same colors as in panel **a**). **c** Model of the 3D structure of the hDAAO (Δ1–16) variant without FAD: the sequences characterized by highest instability are colored in magenta

We next used molecular dynamics simulations to compare the stability of the tertiary structure of full-length (PDB 2E48) with the model of hDAAO (Δ1–16) variant (Fig. 1b), in presence and in absence of the cofactor FAD. Actually, wild-type hDAAO exists in solution in equilibrium between the apoprotein and the holoenzyme forms because of the weak FAD binding [11, 16]. The Root-Mean-Square Deviation (RMSD) during the time of simulation (100 ns) is higher for the Δ1–16 variant compared to the full-length enzyme, in particular in the absence of FAD (Fig. 2a). The Root-Mean-Square Fluctuation (RMSF), calculated to compare the extent of fluctuations of each single residue of the system, is still significantly higher for the hDAAO (Δ1–16) (Fig. 2b). In the absence of FAD, the Δ1–16 variant resulted more unstable than the full-length protein: the regions characterized by highest instability are those located close to the flavin cofactor (residues from 17 to 50, from 135 to 210, and from 260 to 320, Fig. 2c).

Altogether, experimental and in silico analyses suggest that eliminating part of the Rossmann fold [17], and especially of the GXGXXG Wierenga sequence [18], strongly affects the overall fold of the flavoenzyme hampering FAD binding and thus catalytic activity. We concluded that elimination of 16 residues at the *N*-terminal end of hDAAO significantly hampers protein folding, FAD binding, and stability.

### DAAO is present in mouse intestine

The presence of DAAO in tissue fractions from mouse and rat small intestine (luminal, mucosal content, and epithelial layer of proximal, medial, and distal small intestine) was investigated by Western blot analysis using two anti-hDAAO antibodies selected among the five available antibodies because resulted in reproducible results in pilot experiments and could provide information about the lost region, i.e., the non-commercial anti-*N*-terminal and anti-*C*-terminal hDAAO antibodies (from Davids Biotechnologie). The regions recognized by these antibodies are indicated in Fig. 1a. The lack of recognition by the anti-*N*-terminal antibody requires the elimination of 120 residues at the *N* terminus, corresponding to a 13.5 kDa decrease in mass. The elimination of a sequence ≥ 20 residues at the *C* terminus will result in a DAAO form that is not recognized by the anti-*C*-terminal hDAAO antibody.

The analysis of rat intestine samples resulted in nonspecific recognition patterns, see Supplementary Data 3 and Supplementary Table 4. On mice samples, the two antibodies recognize several bands, some of them at a molecular mass higher than intact DAAO, i.e., > 40 kDa. In detail, the main signals recognized by anti-*C*-terminal hDAAO antibodies correspond to bands at 70 kDa in mucosal distal and proximal samples, at 40 kDa in mucosal distal and luminal (proximal, medial, and distal) samples, and at ~ 27 kDa in mucosal proximal and distal samples; faint bands at ~ 100 kDa (in distal mucosal), 70, 45, and ~ 35 kDa (in epithelial layer samples), and at 18 kDa (in proximal and distal mucosal samples) were also detected, see Table 1, Supplementary Data 4 and Supplementary Fig. 2A. The main signals corresponding to anti-*N*-terminal antibody recognition correspond to bands at 40 kDa in distal samples from mucosal and luminal portions (and, with a lower intensity, from proximal and medial samples), at 35 kDa in proximal and medial samples from mucosal and luminal fractions, and at ≤ 20 kDa in epithelial samples (i.e., at 20 and 18 kDa in the medial and at 15 kDa in all samples of the epithelial fraction). Faint bands at 55 kDa (in mucosal samples), 45 kDa (in epithelial layer samples), and 20 kDa (in medial and distal epithelial layer samples) were also detected. The band at ~ 40 kDa, which should correspond to the full-length DAAO, was recognized by both anti-hDAAO antibodies in mucosal distal and luminal medial and distal regions.

**Table 1:** Schematic recognition pattern obtained using different antibodies in Western blot analyses on mouse intestinal tissues (see Suppl. Data 4)

Model	MW (kDa)	*α*-hDAAO-Nterm (DaBio)		*α*-hDAAO-Cterm (DaBio) [*α*-mDAAO-Cterm (Santa Cruz)]	
		Sample	Intensity	Sample	Intensity
Mouse wild type	100			Distal mucosa	*
	70			Proximal mucosa, **medial mucosa**, distal mucosa, epithelial layer (proximal, medial, distal)	**, ****, **, *
	55	**Mucosa** (proximal, **medial**, distal)	*	(Distal mucosa), (proximal mucosa)	(**), (***)
	45	Proximal epithelial, epithelial (medial, distal) **medial mucosa**	**, *	Epithelial layer (proximal, medial, distal)	*
	40	**Distal mucosa**, luminal medial, proximal distal lumen	**, */**, ***	**Distal mucosa**, luminal (proximal, medial, distal)	**, */**
	~ 35–37	**Proximal mucosa**, medial mucosa, proximal lumen, medial lumen	**/***, */**, **, *	Epithelial layer (proximal, medial, distal), (distal, proximal mucosa)	*, (**)
	27	**Medial epithelial layer**		**Proximal mucosa, distal mucosa**	**/***/(**), ***/****/(**)
	20	Proximal epithelial layer, **epithelial layer** (medial, **distal**)	****, *		
	18	Distal epithelial layer	***	Mucosa (proximal, distal)	*
	15	**Epithelial layer** (proximal, **medial**, distal)	*		
Mouse DAAO^−/−^	70			Proximal epithelial	*
	55			(Proximal, distal mucosa)	(**)
	45	Distal mucosa	*	Epithelial layer (proximal, medial, distal)	*
	40	**Distal mucosa**, proximal mucosa	*, *		
	~ 35–37	**Proximal mucosa**, distal mucosa	*, *	Distal mucosa, (proximal mucosa)	***/(*), (*)
	27	Distal mucosa, **proximal epithelial layer**	*, ***	(**Proximal mucosa)**, (**distal mucosa**)	(*), (***)
	18	Mucosa distal	*		
	15	Mucosa distal	*		

**Fig. 3:**
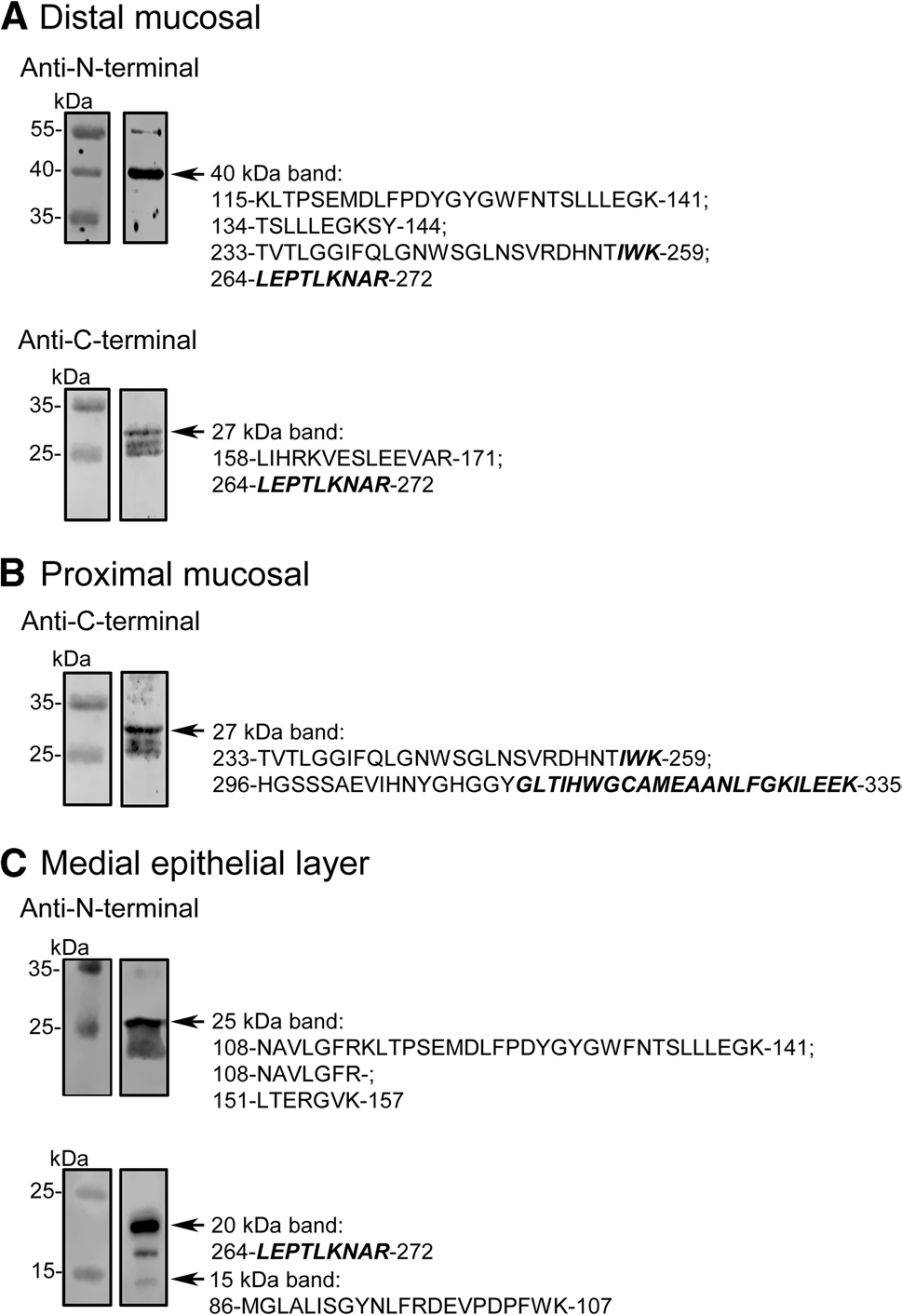
Western blot and MS analysis of distal (**a**) and proximal (**b**) mucosal contents and of medial epithelial layer (**c**) of mouse intestinal samples. The bands recognized by the different antibodies (see arrows) were analyzed by MS/MS for the presence of DAAO: the peptides identified are reported (right side). The sequence belonging to the antimicrobial peptides are shown in bold

The bands indicated in bold have been analyzed by MS: the underlined samples contain DAAO-derived peptides (see Fig. 3). Samples with no asterisk were not recognized by Western blot analysis, but were analyzed by MS

Relative amount: * < 0.1 ng DAAO/µg total proteins; ** 0.1–0.5 ng DAAO/µg total proteins; *** 0.5–1 ng DAAO/µg total proteins; **** > 1 ng DAAO/µg total proteins

To ascertain the specificity of the recognized bands in intestinal tissues, we followed the recommendations recently reported by Mothet et al. [19] by performing control analyses on intestinal tissues isolated from DAAO null constitutive mutant mice (DAAO^−/−^) [20]. Western blot analysis revealed bands at 27 kDa in medial mucosa samples (and faint bands at 45 kDa in epithelial samples, as well as at 70 and 35 kDa in proximal epithelial samples) using the anti-*C*-terminal hDAAO antibody and bands at ~ 27 and 20 kDa in proximal epithelial sample by the anti-*C*-terminal hDAAO antibody (Supplementary Figs. 3 and 4). Similarly, the anti-*C*-terminal mDAAO antibody recognized similar signals in samples isolated from wild-type and DAAO^−/−^ mice (Table 1 and Supplementary Fig. 4A). Altogether, we concluded that available anti-DAAO antibodies recognize also unspecific signals.

The presence of DAAO was thus ascertained by mass spectrometry analysis: the bands at 40 and 27 kDa of the distal mucosa, and the ~ 27 kDa band of the proximal mucosa showed peptides derived from DAAO (three, four, and three, respectively). In the epithelial samples, the 27, 18, and 15 kDa bands of the medial fraction all contained DAAO-derived peptides (Fig. 3 and Supplementary Data 5 and Supplementary Table 5). No peptides deriving from DAAO were identified in the 70 and 45 kDa bands of the medial mucosa and in the ~ 35 kDa band from proximal mucosa, as well as from DAAO^−/−^ mice samples (i.e., the 40 kDa, ~ 35 kDa, and ~ 27 kDa bands from proximal and distal mucosa). Furthermore, mass spectrometry analysis identified DAAO in mucosal distal, medial, and proximal samples from wild-type mice, while no DAAO-derived peptide was identified in the same samples from DAAO^−/−^ mice (Supplementary Table 6).

Altogether, our results indicate that DAAO is released in the lumen of mouse gut, where it is largely proteolyzed. Peptidomic analysis was performed on mucosal samples of wild-type and DAAO^−/−^ mice seeking for the putative 313–345 and 257–289 CAMPs. In wild-type mice, the putative antimicrobial *C*-terminal GLT peptide was detected in mucosal samples, especially in the distal fraction (alternative peptides started from residues 314, 315, 322, and 323), and part of the IWK (the peptide 268–290) in the proximal fraction, Table 2. No peptides originating from DAAO were identified in the DAAO^−/−^ mice.

**Table 2:** DAAO originating peptides identified in mucosal samples from wild-type and DAAO^−/−^ mice

	Wild-type			DAAO^−/−^		
	Proximal	Medial	Distal	Proximal	Medial	Distal
313–345^a^	–	X	X	–	–	–
314–345	–	–	X	–	–	–
315–345 (TIH)	–	–	X	–	–	–
322–345	–	–	X	–	–	–
323–345 (EAA)	–	–	X	–	–	–
268–290^b^ (LKN)	X	–	–	–	–	–

Only peptides identified with high confidence and Xcorr ≥ 1.5 have been considered (indicated with X, while – means not found)

^a^ N326 deamination and M322 oxidation modifications have been considered

^b^ N270 deamination and M322 oxidation modifications have been considered

Although we confirm the secretion of DAAO in the intestinal lumen, some discrepancies are apparent with previously reported observations [11]. We could detect neither the full-length mouse DAAO in the epithelial layer nor the ~ 35–37 kDa protein form lacking the *N*-terminal sequence in the intestinal content. Only bands at ≤ 27 kDa, too small to preserve the enzymatic activity, were instead apparent in both studies. We can not exclude that the use of germ free C57BL/6 mice [11] instead of untreated C57BL/6 mice, as in our case, could have altered the expression of DAAO.

The antimicrobial effect of DAAO in gut was previously related to its catalytic activity [11]. We assayed DAAO activity level by zymograms performed following native PAGE and the sensitive Amplex UltraRed method (Supplementary Data 6). Zymograms on mice intestinal samples showed a faint positive band at low electrophoretic mobility, significantly different from the one observed for the reference recombinant hDAAO and from the signal observed using a mice cerebellum sample (Supplementary Fig. 5A, B). Such an activity signal was also apparent in samples from DAAO^−/−^ mice (Supplementary Fig. 5D, E) or when the substrate d-alanine was omitted from the developing mixture (Supplementary Fig. 5C, F), pointing to aspecific/background signals. The signal recorded using the Amplex UltraRed method is identical with or without the substrate d-alanine or by adding 6-chloro-1,2-benzisoxazol-3(2H)-one (CBIO), an inhibitor of hDAAO [21]: it is close to the detection limit for all tested samples (Supplementary Fig. 6) and four orders of magnitude lower than the value of recombinant hDAAO. In a previous report, DAAO activity was evaluated on intestine tissues by a histological method using a coupled assay with peroxidase and d-proline as substrate [11], a molecule that is also oxidized by alternative enzymes such as d-aspartate oxidase. Most importantly, this latter work identified DAAO activity in epithelial cells only, not in extracellular space. In conclusion, we exclude the presence of active DAAO in mouse gut at a detectable level.

### DAAO-derived CAMPs show antimicrobial activity

Investigating the primary structure of mDAAO using the method described in Pane et al. [13] suggests the presence of two antimicrobial peptides corresponding to 257–289 (IWK) and 315–347 (GLT) regions, see Fig. 1a. Conformation of DAAO antimicrobial peptides was analyzed by far-UV CD. Both the peptides are mostly unstructured and show slightly different spectra (Supplementary Data 7 and Supplementary Fig. 7, black lines). Notably, they assume an ordered helical conformation (minima at around 210 and 220 nm) in the presence of SDS (1 mM) or TFE (30% v/v), two membrane mimicking agents, and of LPS from *E. coli* (0.1 and 0.25 mg/mL for GLT and IWK, respectively) (Supplementary Fig. 7)*.* DichroWeb confirmed the experimental results: both the peptides are initially “unordered” and assume helical conformation during the titration (*α*-helix content > 70%).

To shed light on their putative role in gut microbial selection, the antimicrobial activity of the two polypeptides and a mixture containing both peptides at a 1:1 molar ratio was tested on two Gram-positive and five Gram-negative bacteria (Table 3). Both peptides showed bactericidal or bacteriostatic activity on the tested bacteria, the only exception was *Salmonella enteritidis.* In some cases, an additive effect of the two peptides was apparent reaching MIC values in the low micromolar range. Noteworthy, both peptides showed a strong and additive bacteriostatic activity on *E. faecalis* with the mixture of the two peptides being already active at about 3 μM.

**Table 3:** Antimicrobial activity expressed as MIC and MBC values of two putative CAMPs derived from mouse DAAO on bacteria and lactobacilli

Bacterial strain	Peptide/antibiotic	MIC (μM)	MBC (μM)	MBC/MIC	Bactericidal activity	Bacteriostatic activity	MIC (μg/mL)
Gram-negative							
*Pseudomonas aeruginosa* PAO1	IWK	25	50	2	Yes		
	GLT	100	> 100	–			
	IWK + GLT	12.5	50	4	Yes		
	Polymyxin B						0.25
*Acinetobacter baumanii* ATCC 17878	IWK	12.5	12.5	1	Yes		
	GLT	50	50	1	Yes		
	IWK + GLT	12.5	25	2	Yes		
	Polymyxin B						0.5
*Escherichia coli* ATCC 25922	IWK	12.5	12.5	1	Yes		
	LKN	50	50	1	Yes		
	GLT	25	25	1	Yes		
	TIH	25	25	1	Yes		
	EAA	> 200	–	–			
	IWK + GLT	12.5	100	8		Yes	
	TIH + LKN	12.5	12.5	1	Yes		
	Polymyxin B						0.5
*Salmonella typhimurium* ATCC 14028	IWK	25	50	2	Yes		
	LKN	> 200	–	–			
	GLT	25	> 100	> 4		Yes	
	TIH	25	> 200	> 4		Yes	
	EAA	> 200	–	–			
	IWK + GLT	6.25	100	16		Yes	
	TIH + LKN	25	25	1	Yes		
	Polymyxin B						0.12
*Salmonella enteritidis* 706 RIVM	IWK	100	–	–			
	GLT	100	–	–			
	IWK + GLT	50	–	–			
	Polymyxin B						0.5
Gram-positive							
*Staphylococcus aureus* ATCC 6538P	IWK	25	50	2	Yes		
	GLT	> 100	–	–			
	IWK + GLT	25	50	2	Yes		
	Vancomycin						0.5
*Enterococcus faecalis* ATCC 29212	IWK	12.5	> 100	> 4		Yes	
	LKN	> 200	–	–			
	GLT	6.25	> 100	> 4		Yes	
	TIH	12.5	> 200	> 4		Yes	
	EAA	> 200	–	–			
	IWK + GLT	3.12	> 100	> 4		Yes	
	TIH + LKN	12.5	> 200	> 4		Yes	
	Vancomycin						2

Lactobacilli (MIC, µM)	*L. fermentum* ATCC 14931	*L. casei* ATCC 393	*L. rhamnosus* ATCC 7469	*L. acidophilus* ATCC 4356
IWK	> 110	> 110	> 110	> 110
GLT	> 100	> 100	> 100	> 100
Erythromycin	1.4	2.8	0.7	0.7
Vancomycin	> 100	> 100	> 100	> 100

Four lactic acid bacteria strains belonging to species *Lactobacillus acidophilus*, *L. casei*, *L. fermentum,* and *L. rhamnosus* were tested for their susceptibility to IWK and GLT peptides using broth microdilution (Table 3). The tested strains did not show any significant difference in their antibiotic resistance profile (all strains were resistant to vancomycin and susceptible to erythromycin) and were no sensitive to IWK and GLT peptides in vitro at the highest tested concentration of 100 µM.

For sake of comparison, the antimicrobial activity was also tested on selected Gram-positive and Gram-negative strains using the peptides identified in the mucosal sample, i.e., the 268–290 (LKN), 315–345 (TIH), and 323–345 (EAA) peptides (Tables 2 and 3). The TIH peptide behaved similarly to the GLT peptide, while the EAA shorter version (lacking the *N*-terminal portion) did not show any antibacterial activity. On the other hand, the LKN peptide was less effective than the longer IWK one (residues 257–289) but, when used in combination with the TIH showed a significant additive effect. These results confirm the strong and additive antibacterial activity of peptides generated from DAAO.

## Discussion

DAAO is known to be a peroxisomal enzyme [22–24] even if it was detected in cytosol (i.e., the neo-synthetized protein) [23, 25] and in the nuclei of proximal tubule epithelial cells following treatment with the drug propiverine [26]. Indeed, DAAO was also reported to be secreted in the intestine by goblet cells thanks to the presence of a signal peptide and a predicted cleavage site near the *N* terminus [11]. Interestingly, it was proposed that intestinal processed and active DAAO might control the homeostasis of gut microbiota through the production of H_2_O_2_ using d-amino acids arising from diet and bacteria. Recently, we reported the antimicrobial activity on food specimen of a very active, recombinant DAAO from yeast establishing that it is indeed due to H_2_O_2_ production [27]. Here, we demonstrate that the DAAO form lacking the 16 first amino acid residues is unstable and inactive. Accordingly, any active DAAO species present in the lumen might retain the *N*-terminal sequence required for cofactor binding. Native-PAGE and activity assays excluded the presence of an active DAAO form in mouse gut, in agreement with a recent investigation on DAAO activity in whole intestine as well as proximal, middle, and distal fractions [28]. From Western blot and MS/MS analyses on small intestine mice samples, we concluded that DAAO is present in the epithelial layer and in the mucosal fraction of mouse gut, where it is largely proteolyzed. Altogether, our results exclude H_2_O_2_ generated by DAAO reaction as the mechanism for microbial selection in the gut.

The antimicrobial AS score [29–31] recognized two internal regions in DAAO sequence as CAMPs. The corresponding peptides assumed a secondary structure in the presence of detergents and LPS from *E. coli* and showed antimicrobial activity on various Gram-positive and Gram-negative bacteria but not on Lactobacilli species, which represent the commensal microbiota. The IWK and GLT peptides (and their derivatives TIH and LKN) have been identified in the mucosal fractions. Accordingly, we propose that DAAO selects the gut microbiota through antimicrobial peptides generated by intestinal proteases instead of a direct action based on its catalytic activity and production of H_2_O_2_. An antibacterial activity of DAAO was previously proposed in leukocytes [32, 33]. Our work adds a new function to the physiological roles played by DAAO in mammals, ranging from control on NMDA receptor functionality in the central nervous system acting on d-serine level, to the elimination of d-amino acids in kidney and to the antibacterial activity in neutrophilic leukocytes [5].

**Fig. 4:**
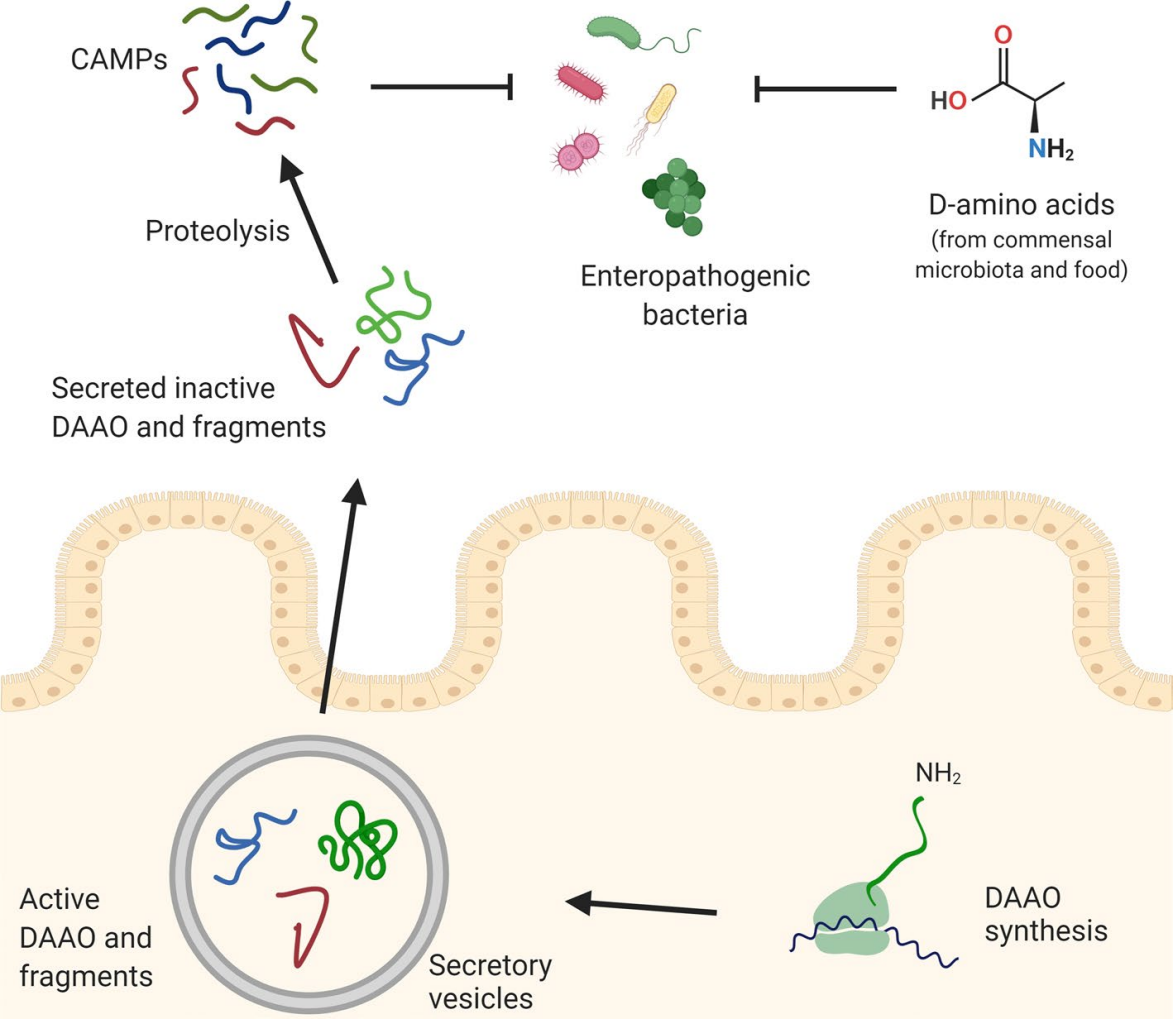
Mode of action proposed for DAAO (produced by epithelial layer cells) in the selection of intestinal microbiota based on antimicrobial peptides generated by proteolytic digestion of the flavoenzyme (starting intracellularly and fulfilled at the extracellular level in the lumen). Microbiota selection can also arise from d-amino acids originating from commensal microbiota and food

Furthermore, since d-amino acids alter the peptidoglycan wall [3] and their level is affected by both nutrients and DAAO activity (as confirmed by the higher d-alanine level observed in DAAO^−/−^ mice) [11], we suggest that DAAO-derived antimicrobial peptides and d-amino acids act independently on microbiota selection in the gut (Fig. 4). An intriguing relationship between gut microbiota and d-amino acids may be potentially identified in neurodegenerative diseases, based on alterations in d-serine and d-glutamate levels in Alzheimer’s disease and other forms of dementia [34, 35].

## Methods

### Molecular dynamics of hDAAO

The 3D structure of the substrate-free form of the human DAAO (hDAAO, PDB 2E48) was used as a starting point for molecular dynamics (MD) simulation performed using the GROMACS package, version 4.6.7. The model of the deleted variant was obtained from the structure of full-length hDAAO through the deletion of the first *N*-terminal 16 residues. For the full-length hDAAO, the system was solvated in a cubic box (volume equal to 670 nm^3^, 8.5 × 7.5 × 10.5 nm) with 20,000 water molecules, while for the deleted variant, a dodecahedral box was used (volume of 610 nm^3^) and solvated with 18,000 water molecules, with the aim to decrease the system dimensions and to increase the rate of simulation. To neutralize the overall charge, an adequate number of Na^+^ ions were added. The system was minimized again with the Steepest Descent method, followed by equilibration of the restrained protein (1000 kJ mol^−1^ nm^−2^ isotropic force applied to each heavy atom of the protein) in the NVT ensemble (up to 200 ps). In each system, the equilibration phase was composed of an inverted simulated annealing starting with an initial temperature of 10 K and rising up to 300 K in 200 ps, keeping the heavy atoms of protein restrained in the same way as in the protein systems. In production phase, each system was simulated for 100 ns in unrestrained conditions in NVT ensemble. Electrostatics was treated with the cut-off method for short-range interactions and with the Particle Mesh Ewald method for the long-range ones (rlist = 1.1 nm, cut-off distance = 1.1 nm, VdW distance = 1.1 nm, PME order = 4). A time step of 2 fs was set using bond-constraining algorithm LINKS. The constant temperature conditions were provided using *V*-rescale thermostat, which is a modification from Berendsen’s coupling algorithm. The AMBER99SB-ILDN force field was used for the simulations.

The Root-Mean-Square Deviation of atomic positions (RMSD, the measure of the average distance between the atoms) was used as a quantitative measure of similarity to compare the stability of the systems (full-length vs. deleted hDAAO variant) in presence or in absence of the flavin cofactor with respect to the initial reference structure. The Root-Mean-Square Fluctuation (RMSF, the measure of the deviation between the positions of a residue averaged over time) was calculated to compare the fluctuations of every single residue in the systems.

### Antibacterial peptides

The sequences of murine and human DAAO were analyzed by an in silico tool [13] which allows to detect the presence and the accurate position of a potential cryptic antimicrobial peptide (CAMP) inside a protein, based on antibacterial “absolute score” (AS) which depends on hydrophobicity, net charge, and length of a peptide. Putative antimicrobial peptides identified inside the sequence of mouse DAAO, ^257^IWKSCCKLEPTLKNARIVGELTGFRPVRPQVRL^289^ (IWK) and ^313^GLTIHWGCAMEAANLFGKILEEKKLSRLPPSHL^345^ (GLT) were synthesized by Aurogene s.r.l. (Roma, Italy), and the ^268^LKNARIVGELTGFRPVRPQVRLE^290^ (LKN), ^323^EAANLGGKILEEKKLSRLPPSHL^345^ (EAA), and ^315^TIHWGCAMEAANLFGKILEEKKLSRLPPSHL^345^ (TIH) by Microtech s.r.l. (Napoli, Italy).

MIC (Minimum Inhibitory Concentration) analyses were performed on Gram-positive and Gram-negative bacteria by a broth microdilution method previously described [13], with minor modifications. In details, assays were carried out in Nutrient Broth 0.5× (Difco, Detroit, MI, USA) using sterile 96-well polypropylene microtiter plates (cat. 3879, Costar Corp., Cambridge, MA, USA). Bacterial strains were grown in Luria–Bertani (LB) medium overnight at 37 °C and then diluted in nutrient broth at a final concentration of ~ 5 × 10^5^ CFU/mL per well. IWK and GLT peptides were suspended in 5 mM sodium acetate buffer, pH 5. Peptide concentrations were determined by spectrophotometric analysis using the extinction coefficients calculated by the ProtParam tool (http://web.expasy.org/protparam/). Twofold serial dilutions of peptides were carried out in the test wells (0.2–100 μM concentration range). Plates were incubated overnight at 37 °C. MIC value was taken as the lowest concentration at which growth was inhibited. Three independent experiments were performed for each MIC value. The peptide antibiotic polymyxin B and vancomycin (Sigma, St. Louis, MO) were tested as control. MIC values were measured on: *Pseudomonas aeruginosa* PAO1, *Acinetobacter baumanii* ATCC 17878, *Escherichia coli* ATCC 25922, *Salmonella typhimurium* ATCC 14028, *Salmonella enteritidis* 706 RIVM (kindly provided by Prof. E. Veldhuizen, Utrecht University, Holland), *Staphylococcus aureus* ATCC 6538P, and *Enterococcus faecalis* ATCC 29212.

The Minimum Bactericidal Concentration (MBC) was determined from the broth dilution of MIC tests by subculturing cell mixtures on agar plates. The MBC was defined as the lowest concentration of antibacterial agent that kills ≥ 99.9% of bacterial cells. According to a widely accepted criterion, peptides were considered bactericidal if their MBC was no more than four times the MIC.

To assess the effect of antibacterial peptides on lactobacilli, *Lactobacillus acidophilus* ATCC 4356, *Lactobacillus casei* ATCC 393, *Lactobacillus fermentum* ATCC 14931, and *Lactobacillus rhamnosus* ATCC 7469 strains (from American Type Culture Collection) were used. Before performing the test, cultures were streaked on Lactobacilli MRS agar (MRS) and incubated for 24 h at 37 °C in an anaerobic chamber (BD Difco, Detroit, MI, USA). Antibacterial susceptibility testing was performed according to the ISO 10932/IDF 233 standard (ISO, 2010). Erythromycin and vancomycin (Sigma-Aldrich, USA) stock solutions were prepared at a concentration of 1 mM, and IWK and GLT peptides at 400 µM. Typically, a twofold serial dilution of stock solutions was performed in Lactobacilli MRS broth to obtain solutions in the 0.2–100 µM concentration range. Bacterial inocula were prepared by suspending colonies, from 24 h incubated Lactobacilli MRS agar plates, into 2 mL of 0.85% NaCl solution. Subsequently, inocula were adjusted to have an OD_625 nm_ of 0.25 and diluted 1:200 in Lactobacilli MRS broth for inoculation of microdilution plates by adding 50 µL of diluted inoculum to each well containing 50 µL of an antibiotic/peptide solution (the bacterial inoculum was around 3–8 × 10^5^ CFU/mL in the wells). MIC values were estimated after incubating the plates under anaerobic conditions at 37 °C for 48 h.

### Preparation of hDAAO (Δ1–16) deletion variant in *Escherichia coli*

The cDNA encoding the *N*-terminal deleted variant lacking the secretion signal peptide, namely hDAAO (Δ1–16), was prepared by PCR using the pET11b-His-hDAAO plasmid, as detailed in Supplemental Data 2. For the co-expression of hDAAO (Δ1–16) variant with the His-tagged full-length hDAAO, the pET-Duet-1 vector (Novagen, Darmstadt, Germany) was used. Recombinant hDAAO wild-type and deleted variants were expressed in BL21(DE3)Star *E. coli* cells and purified as reported in Ref. [14]; 40 µM of free FAD was present during all purification steps. The co-expressed deleted variant and full-length hDAAO (which were expected to produce heterodimers) were purified using the protocol setup for the His-tagged hDAAO (by HiTrap chelating chromatography) or the protocol suggested for the purification of strep-tagged protein (by strep-trap HP chromatography) for the strep-tagged hDAAO (Δ1–16). DAAO activity was assayed with an oxygen electrode at pH 8.5, air saturation, and 25 °C, using 28 mM d-alanine as substrate in the presence of 0.2 mM FAD [14].

### Isolation of luminal, mucosal, and epithelial layers in the small intestine

Slightly modified version of the protocol described in Vaishnava et al. [36] was used to isolate luminal and mucosal layers in the small intestine of Wistar rats (2 males and 2 females, 2–3 months old) and C57BL/6 J mice (2 males and 1 female, 2–3 months old). The protocol reported in Ref. [37] was followed for isolation of the epithelial layer. Freshly dissected rat or mouse small intestines were partitioned into three regions: proximal, medial, and distal samples. Then each piece was further subdivided into three parts: (i) the ‘luminal content’ corresponded to the washing out with 2 mL of ice-cold sterile phosphate buffer saline (PBS); (ii) the tissue pieces were opened longitudinally and placed in 15 mL tubes with 2 mL of ice-cold sterilized PBS. Tubes were inverted 20 times and vortexed for 10 s: the collected liquid represented the ‘mucosal content’; (iii) finally, the ‘epithelial layer’ was recovered: the remaining tissue pieces were cut into 3–5 mm square patches, mixed with 10 mL of PBS containing 0.5 mM DTT and 5 mM EDTA, and incubated with constant shaking at 37 °C for 20 min. Epithelial cells were recovered by pipetting for ten times and filtering the sample through a 70 μm cell strainer to remove tissue fragments; cells were pelleted by centrifugation at 6000 g at 4 °C for 10 min [11]. As negative control, DAAO^−/−^ knock-out mice which do not express the flavoenzyme were used [20].

### Western blot analyses

The presence of DAAO in the different gut samples was investigated by Western blot analyses using different antibodies: anti-hDAAO, diluted 1:3000 (Davids Biotechnologie, Regensburg, Germany); anti-hDAAO *C*-terminal, diluted 1:3000 (Davids Biotechnologie); anti-hDAAO *N*-terminal, diluted 1:250 (Davids Biotechnologie); anti-hDAAO diluted 1:1000 (Abcam, Cambridge, UK); anti-hDAAO, diluted 1:500 (Rockland, Limerick, PA, USA); anti-mDAAO *C*-terminal, diluted 1:500 (Santa Cruz Biotechnology, Dallas, TX, USA). Epithelial cells were homogenized in lysis buffer (150 mM sodium chloride, 1% NP-40, 50 mM Tris, pH 8.0, and a proteases’ inhibitor cocktail), and luminal and mucosal contents isolated from the small intestine were sonicated and centrifuged. Protein level in supernatants was quantified using Bradford reagent. A cerebellar lysate was used as a positive control for the presence of DAAO. Supernatants were subjected to SDS-PAGE and proteins were transferred to PVDF membranes. Western blot analyses were performed as reported in Ref. [23]; densitometric analysis was performed to assess the amount of protein in the detected bands.

### nLC-MS/MS analysis

To verify the presence of DAAO in the small intestine, tissue samples were analyzed by mass spectrometry. 160 µg (40 × 4 lanes) of total proteins from different gut fractions were separated by SDS-PAGE and stained with colloidal Coomassie; the bands of interest (i.e., at the selected molecular mass values) were cut and stored in 5% acetic acid for the identification by nLC-MS/MS. Each band was cut and digested in situ by trypsin sequence grade upon extraction with trichloroacetic acid and acetonitrile, reduction with 45 mM dithiothreitol, and alkylation with 100 mM iodoacetamide. MS/MS analysis was carried out by an LTQ-Orbitrap Velos (Thermo Fisher Sci., Waltham, MA, USA), as previously described [38]. Database search was performed using the Sequest search engine of Proteome Discoverer 1.4 (Thermo Fisher Sci.) against the Rodentia and OXDA mouse (P18894) Uniprot sequence databases. Only peptides with Xcorr ≥ 1.45 and Confidence FDR ≤ 0.05 were kept for the identification.

To confirm the presence of peptides originated from mouse DAAO, mucosal contents of proximal, medial, and distal small intestine of C57BL/6 J and C57BL/6 J DAAO^−/−^ knock-out mice were analyzed by a mass spectrometry-based peptidomic approach. Samples were precipitated with two volumes of cold acetonitrile containing 0.1% of trifluoroacetic acid (stored at − 20 °C overnight) and centrifuged at 13,200 rpm for 30 min to remove proteins. The supernatant containing peptides and low-molecular-weight proteins were collected, dried (Speed Vacuum), dissolved in 1% (v/v) formic acid, and desalted (Zip-Tip C18, Millipore) before mass spectrometric (MS) analysis. MS/MS analysis was carried out by a Orbitrap Fusion Tribrid mass spectrometer (Thermo Fisher Sci., Waltham, MA, USA), as previously described [39]. Data Base search was performed using the Sequest search engine of Proteome Discoverer 1.4 (Thermo Fisher Sci.) against OXDA mouse (P18894) Uniprot sequence database. Only peptides with Xcorr ≥ 1.5 and high confidence were kept for the identification.

### DAAO activity of intestinal samples

With the aim to verify the presence of active DAAO in the intestinal fractions, two methods have been used: (a) native PAGE: gels were stained for DAAO activity based on the reduction of iodonitrotetrazolium salt, i.e., by incubating at 37 °C, the gel in 35 mM sodium pyrophosphate, pH 8.5, 0.2 mM FAD, 28 mM d-Ala, and 0.18 mM iodonitrotetrazolium dissolved in ethanol [40]; (b) Amplex UltraRed (Thermo Fisher Sci.) detecting hydrogen peroxide formation [23]. Controls were performed by adding the DAAO specific inhibitor CBIO or removing the substrate in the assay solution (negative controls), as well as using the recombinant hDAAO or mice cerebellum samples (positive controls).

### Supplementary Information

Below is the link to the electronic supplementary material.

Supplementary file1 (DOCX 1823 KB)

## Data Availability

The datasets generated during and/or analysed during the current study are available from the corresponding author on reasonable request.
