# Supplementary material for: Antimicrobial d-amino acid oxidase-derived peptides specify gut microbiota
Source: Cell Mol Life Sci. 2021 Jan 23;78(7):3607–20. doi: 10.1007/s00018-020-03755-w (PMC8038955; doi:10.1007/s00018-020-03755-w)
Supplement: Supplementary file 1 — Supplementary file1 (DOCX 1823 KB) [file 18_2020_3755_MOESM1_ESM.docx]

**Antimicrobial D-amino acid oxidase-derived peptides specify gut microbiota**

Giulia Murtas^a^, Silvia Sacchi^a,b^, Gabriella Tedeschi^c,d^, Elisa Maffioli^c,d^, Eugenio Notomista^e^, Valeria Cafaro^e^, Monica Abbondi^b,f^, Jean-Pierre Mothet^g^, Loredano Pollegioni^a*^

^a^ Department of Biotechnology and Life Sciences, University of Insubria, Varese, Italy

^b^ DAAIR, D-Amino Acid International Research Center, Gerenzano, Italy

^c^ Department of Veterinary Medicine, University of Milan, Milan, Italy

^c^ Cimaina, University of Milan, Milan, Italy

^e^ Department of Biology, University of Naples Federico II, Naples, Italy

^f^ Fondazione Istituto Insubrico Ricerca per la Vita (FIIRV), Gerenzano, Italy

^g^ LuMIn FRE2036, Biophotonics and Synapse Physiopathology Team, Université Paris-Saclay, CNRS, ENS Paris-Saclay, CentraleSupélec, 91190, Gif-sur-Yvette, France

^*^Corresponding author:

Loredano Pollegioni, Department of Biotechnology and Life Sciences, University of Insubria,

Via J. H. Dunant 3, 21100 Varese, Italy. e-mail address: loredano.pollegioni@uninsubria.it

Tel: +39 0332 421506 - Fax: +39 0332 421 500.

Authors’ e-mail addresses: g.murtas@uninsubria.it; silvia.sacchi@unisubria.it; gabriella.tedeschi@unimi.it; elisa.maffioli@unimi.it; eugenio.notomista@unina.it; valeria.cafaro@unina.it; monicaabbondi@ricercaperlavita.it; jean-pierre.mothet@u-psud.fr; loredano.pollegioni@uninsubria.it

Authors’ ORCID: 0000-0002-9086-5520 (GM); 0000-0002-2338-2561 (SS); 0000-0003-2082-6443 (GT); 0000-0002-5539-1825 (EM); 0000-0003-0097-6487 (EN); 0000-0002-6686-7657 (VC); 0000-0002-8248-2861 (MA); 0000-0003-4991-0614 (JPM); 0000-0003-1733-7243 (LP).

**This file includes:**

Supplementary Data 1-7

Supplementary Figures 1-7

Supplementary Tables 1-5

**Supplemental Data 1. *In silico* analysis of DAAO sequences**

The sequences of mammalian DAAOs (from human, mouse, rat and porcine) were analyzed for the presence of a signal peptide and a cleavage site for protein secretion with different bioinformatics servers: i) SignalP 3.0 Server, which predicts the presence and location of signal peptide cleavage sites in amino acid sequences based on a combination of several artificial neural networks and hidden Markov models [1]; ii) SignalP 4.1 Server, an improved version of the previous one, which does not consider the hidden Markov model and is able to discriminate between signal peptides and transmembrane regions [2]; iii) SecretomeP 2.0 Server, which produces *ab initio* predictions of non-classical protein secretion. The method queries many other feature prediction servers to obtain information on various post-translational and localization aspects of the protein, which are integrated into the final secretion prediction [3]; iv) Signal-3L server, which determines the presence of a signal peptide and cleavage site and selects a final unique site in concertation with its evolution conservation score [4]; v) Signal-3L 2.0 Server, an improved version of the previous one [5].

**Supplementary Table 1:** Prediction of N-terminal signal sequence for secretion in DAAO from different sources.

|  | **SignalP 3.0** | **SignalP 4.1** | **SecretomeP 2.0** | **Signal-3L** | **Signal-3L 2.0** |
| --- | --- | --- | --- | --- | --- |
| **Human** | 0.749 | 0.447 | 0.782 | yes | no |
| **Mouse** | 0.700 | 0.415 | 0.693 | yes | yes |
| **Rat** | 0.699 | 0.415 | 0.685 | yes | yes |
| **Porcine** | 0.756 | 0.455 | 0.667 | yes | no |

Cutoff values: 0.430 for SignalP 3.0; 0.450 for SignalP 4.1; 0.600 for SecretomeP 2.0

**Supplementary Fig. 1.** Sliding window analysis of mouse DAAO sequence for identification of antibacterial sequences. Absolute score (AS) values were calculated using parameters optimized for *S. aureus* strain C623 and the hydrophobicity scale “Parker-Arg0”. Plots were obtained using window lengths of 22 (blue line) and 33 residues (red line). Dotted lines indicate AS values corresponding approximately to hypothetical MIC values of 100 (bottom), 10 and 1 (top) μM.

**Supplementary Data 2. Production of hDAAO(Δ1-16)**

**Preparation and expression of hDAAO deletion variant in *Escherichia coli***

The cDNA encoding the N-terminal deleted variant lacking the secretion signal peptide, namely hDAAO(Δ1-16), was prepared by PCR using the pET11b-His-hDAAO plasmid as the template and the following primers: 5’-CACCATATGCTCTGCATCCATGAGCGC-3’ (annealing downstream to the canonical starting codon, starting from the codon for Leu17 and containing the *Nde*I restriction site, underlined) and 3’-ATGGATCCTCT**TCA**GAGGTGGGATGGTG-5’ (annealing downstream the stop codon, in bold, and containing the *BamH*I restriction site, underlined). The amplified insert was then subcloned in the pET11b plasmid.

For the co-expression of the N-terminal deleted variant with the His-tagged full-length hDAAO, the pET-Duet-1 vector (Novagen, Darmstadt, Germany) was used. The His-hDAAO wild-type [6] was subcloned into the multiple cloning site 1; the PCR reaction was carried out using the pET11b-His-hDAAOwt as template and the following primers: 5’-cacCCATGG**ATG**CATCACCATCACCATCACATG-3’ (annealing downstream to the canonical starting codon of the His-hDAAO wild-type, in bold, and adding a *Nco*I restriction site, underlined) and the 3’-atAAGCTTTCT**TCA**GAGGTGGGATGGTGGCAT-5’ (annealing downstream to the canonical stop codon, in bold, and adding a *Hind*III restriction site, underlined). The amplified fragment was inserted into the pET-Duet vector between the *Nco*I-*Hind*III sites. The deleted strep-tagged hDAAO(Δ1-16) variant was subcloned into the multiple cloning site 2; the gene was amplified using the pET11b-hDAAO(Δ1-16) as a template using the following primers: 5’-cacCAT**ATG**CTCTGCATCCATGAGCGCTAC-3’ (annealing downstream to the canonical starting codon of hDAAO(Δ1-16), in bold, and adding a *Nde*I restriction site, underlined) and 3’-atCTCGAGTCT**TCA***CTTTTCGAACTGCGGGTGGCTCCA*GAGGTGGGATGGTGGCATTCTGGA-5’ (annealing downstream to the canonical stop codon, in bold, and adding a *Xho*I restriction site, underlined, and the strep-tag, italic). The amplified fragment was inserted into the pET-Duet vector between *Nde*I and *Xho*I sites.

Recombinant hDAAO wild-type and deleted variant were expressed in BL21(DE3)Star *E. coli* cells and purified as reported in [6, 7] (Supplementary Table 2)*;* 40 µM of free FAD was present during all purification steps. The deleted variant was purified by using the procedure previously set up for the untagged wild-type enzyme consisting of ammonium sulfate precipitation at 35% saturation, followed by dialysis of the pellet, and anionic exchange chromatography on DEAE Sepharose FF at pH 8.0 (as well as using different resins and buffers).

The co-expressed deleted variant and full-length hDAAO (which were expected to produce heterodimers) were purified using the same protocol set up for the His-tagged wild-type enzyme (by HiTrap Chelating chromatography) [6] or the protocol suggested for the purification of strep-tagged protein (Strep-Trap HP chromatography, in TrisHCl pH 8.0 or PBS pH 7.4 using desthiobiotin in the elution buffer) for the strep-tagged hDAAO(Δ1-16). No DAAO enzymatic activity was detected in the crude extract of BL21(DE3)pLysS *E. coli* cells harbouring the pET-Duet vector. Neither a HiTrap chelating chromatography nor a StrepTrap column (using desthiobiotin in the elution buffer) allowed binding of the recombinant proteins: in both cases, the two protein forms eluted in the flow-through. None of the conditions reported in Supplementary Table 3 allowed to produce a suitable amount of hDAAO(Δ1-16): the recombinant protein poorly binds to different resins and appears highly unstable and inactive.

The final enzyme preparations were stored in 20 mM Tris-HCl buffer, pH 8.0, 100 mM NaCl, 10% (v/v) glycerol, 40 µM FAD and 5 mM 2-mercaptoethanol. The enzyme concentration was determined spectrophotometrically by using the extinction coefficient at 445 nm (12.2 mM^-1^ cm^-1^). DAAO activity was assayed with an oxygen electrode at pH 8.5, air saturation and 25 °C, using 28 mM D-alanine as substrate in the presence of 0.2 mM FAD [6].

**Supplementary Table 2:** Comparison of the best fermentation conditions used for the expression of wild-type and deleted variant of hDAAO (and for co-expression).

| **Conditions** | **hDAAO variant** | | |
| --- | --- | --- | --- |
|  | **Δ1-16** | **Δ1-16/wild-type** | **wild-type** |
| *E. coli* Strain | BL21(DE3)Star | BL21(DE3)pLysS | BL21(DE3)Star |
| Cultivation broth | TB | LB | TB |
| IPTG (mM) | 0.1 | 1 | 0.1 |
| Temperature after induction (°C) | 25 | 37 | 37 |
| Time of cell collection after induction (h) | 6 | 3 | 4 |
| OD_600nm_ at induction | 0.8 | 0.6 | 8 |
| Expression level (mg/L) in the crude extract | 2.1 | 0.8 | 7 |

**Supplementary Table 3:** Purification trials for hDAAO(Δ1-16) and for His-hDAAO wild-type/strept-hDAAO(Δ1-16).

|  | **pET11b-hDAAO(Δ1-16)**  **(no tag)** | **pETDuet-His-hDAAO wild-type/strep-hDAAO(Δ1-16)** |
| --- | --- | --- |
| Chromatography | Ion-exchange | Affinity |
| Column/Resin | Anionic and cationic (DEAE, SP FF, Q FF) | HiTrap chelating (Ni^2+^), Strep-trap |
| Buffer | Tris, BisTris-propane, MES | Na pyrosphosphate, PBS, Tris |
| pH | 6, 8, 8.7, 9.2 | 7.2, 7.4, 8.0 |

**Supplementary Data 3. Western blot analyses of rat intestine samples**

As summarized in Supplementary Table 4, different bands were recognized by non-commercial anti-N-terminal and anti-C-terminal hDAAO antibodies in rat tissues. In details, two bands at a molecular mass higher than wild-type DAAO have been recognized by the anti-N-terminal hDAAO antibody, namely at 100 and 55 kDa, which are apparent in both mucosal and luminal layers: these bands most likely should correspond to aspecific signals. Both the antibodies recognized a band at 40 kDa, but in different samples. Indeed, the anti-N-terminal antibody recognized bands at 35, 25 and ~ 10 kDa while the anti-C-terminal antibody recognized bands at 30 and 20 kDa that should derive from elimination of an N-terminal region. Notably, although the > 25 kDa mass fragments should be recognized by both antibodies, this was not observed. Altogether, the employed antibodies seem to detect aspecific signals in rat intestinal samples.

**Supplementary Table 4:** Schematic recognition pattern obtained using different antibodies in Western blot analyses on rat intestinal tissues. EP = epithelial layer.

| **MW (kDa)** | **α-hDAAO-Nterm (DaBio)** | | |  | **α-hDAAO-Cterm (DaBio)** | | |
| --- | --- | --- | --- | --- | --- | --- | --- |
| **100** | mucosa (proximal, medium, distal)  proximal lumen  medial lumen | **  ***  * |  | |  |  |  |
| **55** | distal mucosa  proximal mucosa | **  *** |  | |  |  |  |
| **~ 40** | mucosa (proximal, medial, distal) | **/*** |  | | medial EP layer | * |  |
| **~ 35** | distal lumen | ** |  | |  |  |  |
| **30** |  |  |  | | mucosa (proximal, medium, distal)  distal lumen  distal EP layer | **  *  * |  |
| **25** | distal mucosa  proximal mucosa | **  ** |  | |  |  |  |
| **20** |  |  |  | | medial mucosa | ** |  |
| **~ 10** | proximal mucosa  proximal lumen  medial lumen | **  ****  ** |  | |  |  |  |

Relative amount: * < 0.1 ng DAAO/µg total proteins; ** 0.1-0.5 ng DAAO/µg total proteins; *** 0.5-1 ng DAAO/µg total proteins; **** > 1 ng DAAO/µg total proteins

**Supplementary Data 4. Western blot analyses of mouse intestine samples**

On mice samples, the main signals recognized by anti-C-terminal hDAAO antibodies correspond to bands at 70 kDa in distal and proximal mucosa, at 40 kDa in distal and luminal mucosa (proximal, medial and distal SI) samples, and at ~ 27 kDa in proximal and distal mucosa, see Table 1 and Supplementary Fig. 2A. The band at ~ 40 kDa, that should correspond to the full-length DAAO, was recognized by both anti-hDAAO antibodies in distal mucosa and medial and distal lumen. Faint bands at ~ 100 kDa (in distal mucosa), 70, 45 and ~ 35 kDa (in epithelial layer samples), and at 18 kDa (in proximal and distal mucosa) were also detected depending on sample concentration and preparation (see the samples indicated by a single asterisk in Table 1). The anti-C-terminal mDAAO antibody recognized bands at 55, 35 and 27 kDa in proximal and distal mucosa: the band at lower molecular mass was also recognized by the anti-C-terminal hDAAO antibody (see Table 1 and Supplementary Fig. 3).

The main signals corresponding to anti-N-terminal antibody recognition corresponds to bands at 40 kDa in distal samples from mucosal and luminal portions (and, with a lower intensity, from proximal and medial samples), at 35 kDa in proximal and medial samples from mucosal and luminal fractions, and at ≤ 20 kDa in epithelial samples (i.e., at 20 and 18 kDa in the medial and at 15 kDa in all samples of the epithelial fraction). These results should arise from a specific recognition since the apparent mass values agree with protein fragments harboring the regions recognized by the two antibodies. Notably, bands at ≤ 20 kDa are recognized only in the epithelial layer: the 20 kDa-band in proximal epithelial layer corresponds to 5.7 ng / µg total proteins. Faint bands at 55 kDa (in mucosal samples), 45 kDa (in epithelial layer samples), 20 kDa (in medial and distal epithelial layer samples) were also detected based on sample concentration and preparation (see the samples indicated by a single asterisk in Table 1).

In order to ascertain the specificity of the recognized bands in intestinal tissues, we next performed control analyses on intestinal tissues isolated from DAAO null constitutive mutant mice (DAAO^-/-^). Western blot analysis revealed bands at 27 kDa in medial mucosa samples (and faint bands at 45 kDa in epithelial samples, as well as at 70 and 35 kDa in proximal epithelial samples) using the anti-C-terminal hDAAO antibody and a band at ~ 27 and 20 kDa in proximal epithelial sample by the anti-C-terminal hDAAO antibody (Supplementary Data 4 and Supplementary Figs. 3 and 4). The anti-C-terminal mDAAO antibody recognized similar signals in samples isolated from wild-type and DAAO^-/-^ mice (Table 1 and Supplementary Fig. 4A).


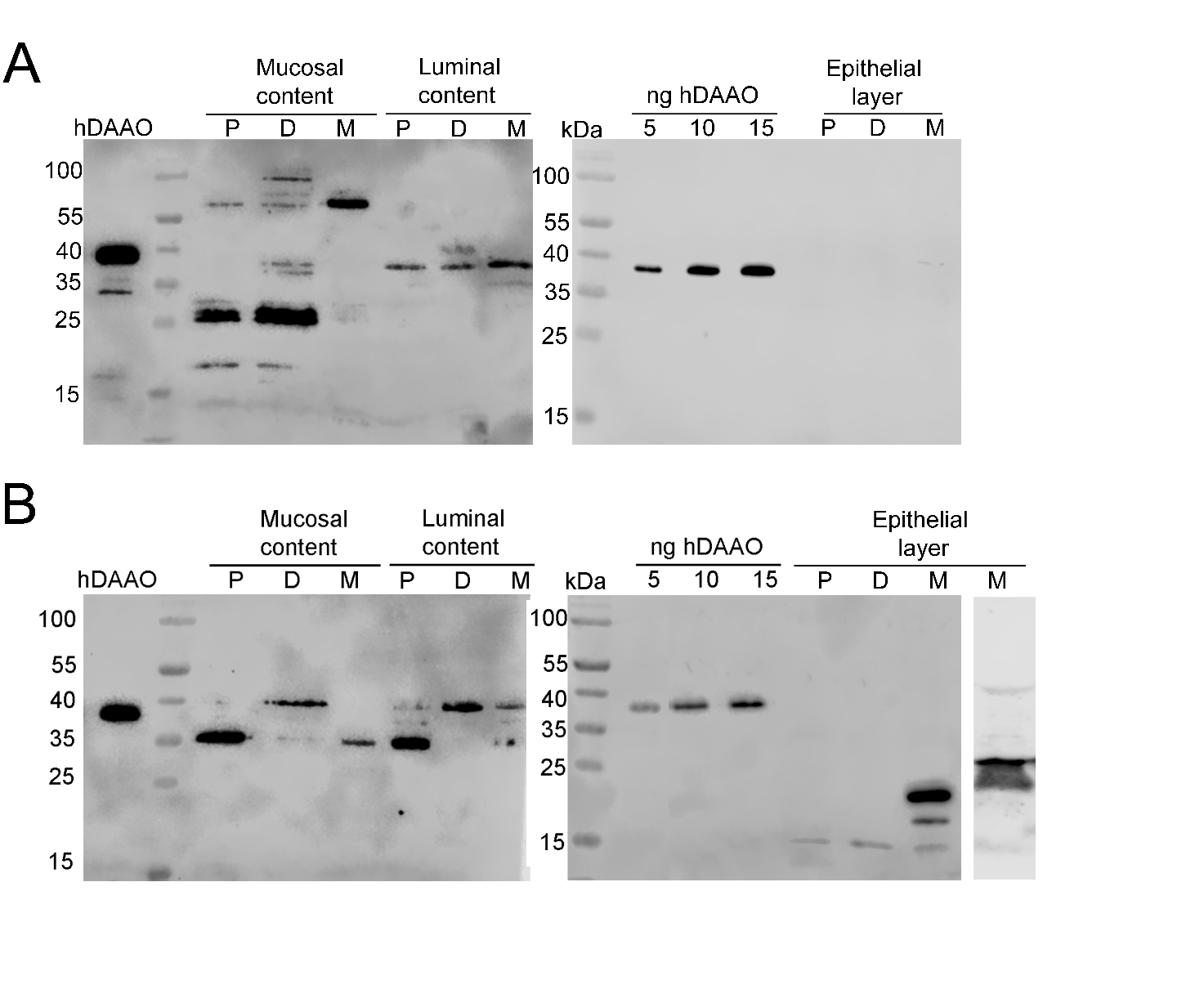


**Supplementary Fig. 2.** Western blot analysis of mouse intestine samples using anti-C-terminal (panel A) or anti-N-terminal hDAAO antibodies (panel B). In each lane an amount corresponding to 30 µg of total proteins was loaded. Standard hDAAO in the left panels: 15 ng. P = proximal, M = medial, D = distal regions of mouse intestine.


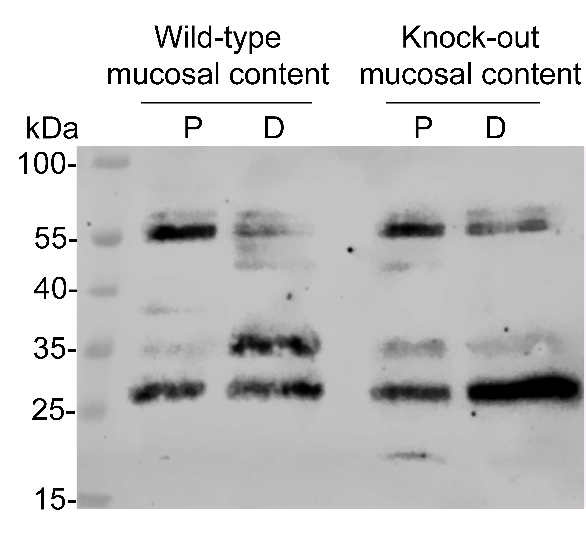


**Supplementary Fig. 3.** Western blot analysis of proximal and distal mucosal samples from wild-type or DAAO^-/-^ mice using an anti-C-terminal mDAAO antibody. In each lane an amount corresponding to 40 µg of total proteins was loaded. P = proximal, D = distal regions of mouse intestine.


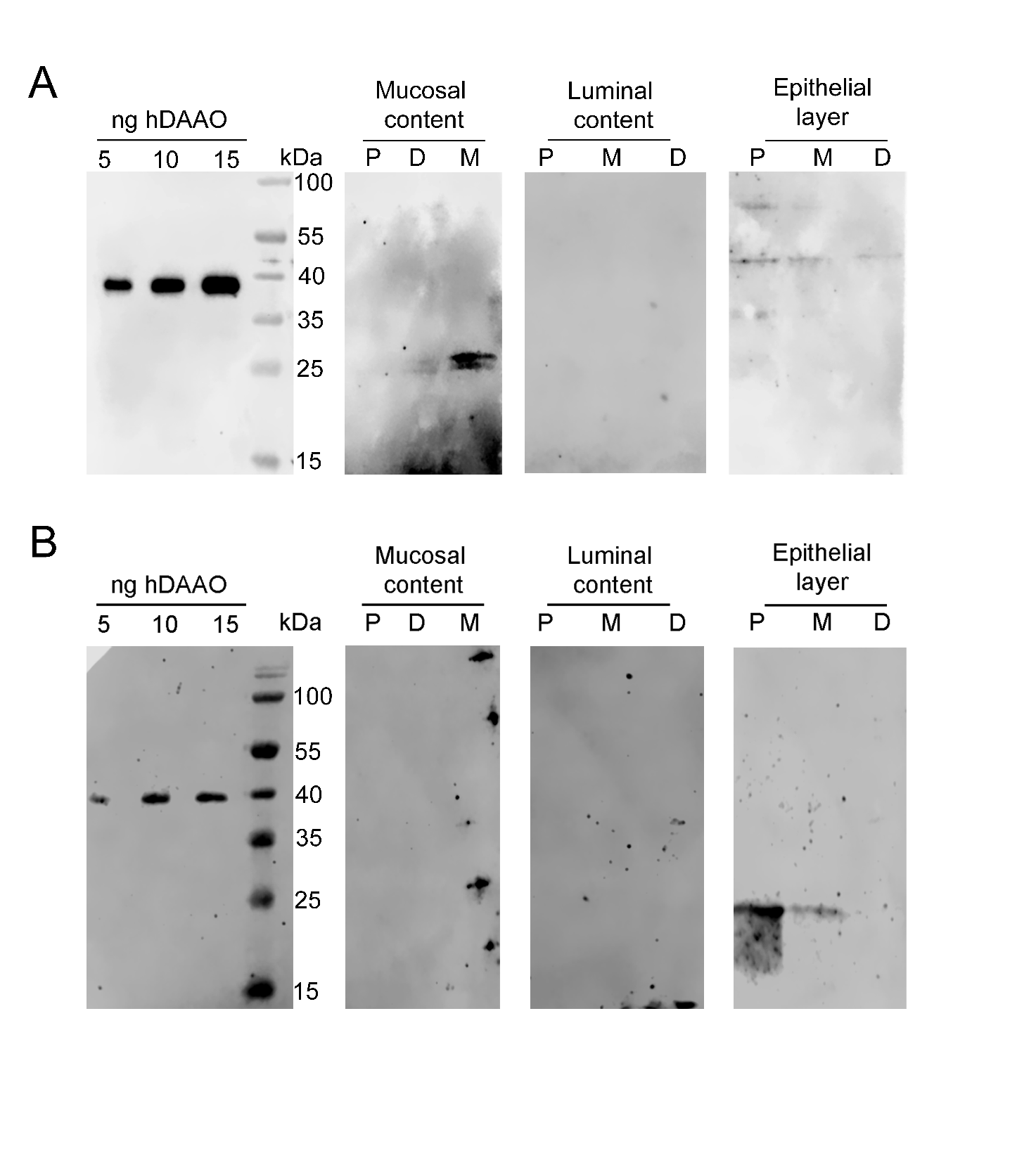


**Supplementary Fig. 4.** Western blot analysis of DAAO^-/-^ mouse intestine samples using anti-C-terminal (panel A) or anti-N-terminal hDAAO antibodies (panel B). In each lane an amount corresponding to 40 µg of total proteins was loaded. P = proximal, M = medial, D = distal regions of mouse intestine.

**Supplementary Data 5. MS/MS analysis of distal and proximal mucosal content of mouse intestinal samples**

The bands recognized by anti-N-terminal and anti-C-terminal hDAAO antibodies in mouse tissues were analyzed by MS/MS in order to identify the proteins present. Supplementary Table 5 reports the list of the peptides of DAAO identified with medium (XCorr ≥ 1.0) and high confidence (XCorr ≥ 1.45). The numbers at the N- and C-terminus of each peptide indicate the position in the sequence. Modified amino acids are reported in lowercase letters.

Supplementary Table 6 reports the peptides of DAAO identified by mass spectroscopy on proximal, medial and distal mucosal samples from wild-type and DAAO^-/-^ mice.

**Supplementary Table 5.** List of the peptides identified by mass spectrometry on different mice intestine samples.

| **Proximal mucosa ~27 kDa band:** | | |  |  | |  | |  | |  | |  | |
| --- | --- | --- | --- | --- | --- | --- | --- | --- | --- | --- | --- | --- | --- |
|  | | **Sequence** | **Modifications** | **XCorr** | **Charge** | **MH+ [Da]** | | **ΔM [ppm]** | | **RT [min]** | | **Missed Cleavages** | |
|  | | 198-GqIIQVEAPWIK-209 | Q199(Deamidated) | 1.04 | 2 | 1382.76299 | | -3.55 | | 67.58 | | 0 | |
|  | | 296-HGSSSAEVIHNYGHGGYGLTIHWGcAmEAANLFGKILEEK-335 | C320(Carbamidomethyl); M322(Oxidation) | **1.81** | 4 | 4357.09487 | | 9.03 | | 92.60 | | 1 | |
|  | | 296-HGSSSAEVIHNYGHGGYGLTIHWGcAmEAANLFGKILEEK-335 | C320(Carbamidomethyl); M322(Oxidation) | **1.81** | 4 | 4357.09487 | | 9.03 | | 92.60 | | 1 | |
|  | | 115-KLTPSEMDLFPDYGYGWFNTSLLLEGK-141 |  | 1.19 | 3 | 3121.55271 | | 9.57 | | 60.02 | | 1 | |
|  | | 158-LIHRKVESLEEVAR-171 |  | 1.11 | 3 | 1678.97202 | | 7.18 | | 70.12 | | 2 | |
|  | | 158-LIHRKVESLEEVAR-171 |  | 1.04 | 3 | 1678.94822 | | -7.00 | | 56.71 | | 2 | |
|  | | 151-LTERGVK-157 |  | 1.08 | 2 | 802.47148 | | -8.33 | | 7.47 | | 1 | |
|  | | 86-MGLALISGYNLFR-98 |  | 1.49 | 3 | 1454.79609 | | 9.34 | | 16.52 | | 0 | |
|  | | 152-SYLPWLTERLTERGVK-157 |  | 1.12 | 4 | 1948.04751 | | -9.06 | | 58.97 | | 2 | |
|  | | 233-TVTLGGIFQLGNWSGLnSVRDHNTIWK-259 | N249(Deamidated) | **1.50** | 3 | 3014.57370 | | 8.39 | | 104.95 | | 1 | |
|  | | 233-TVTLGGIFQLGNWSGLnSVRDHNTIWK-259 | N249(Deamidated) | **1.50** | 3 | 3014.57370 | | 8.39 | | 104.95 | | 1 | |
|  | | 233-TVTLGGIFQLGnWSGLnSVRDHNTIWK-259 | N244(Deamidated); N249(Deamidated) | 1.39 | 3 | 3015.54673 | | 4.74 | | 104.98 | | 1 | |
|  | | 233-TVTLGGIFQLGNWSGLnSVRDHNTIWK-259 |  | 1.26 | 3 | 3013.56314 | | -0.41 | | 104.91 | | 1 | |
|  | | 23-YHPTQPLHmK-32 | M31(Oxidation) | 1.00 | 2 | 1267.62273 | | -1.98 | | 50.16 | | 0 | |
|  | |  |  |  |  |  | |  | |  | |  | |
| **Distal mucosa ~ 27 kDa band:** | | | |  |  |  | |  | |  | |  | |
|  | | **Sequence** | **Modifications** | **XCorr** | **Charge** | **MH+ [Da]** | | **ΔM [ppm]** | | **RT [min]** | | **Missed Cleavages** | |
|  | | 158-LIHRKVESLEEVAR-171 |  | **1.76** | 3 | 1678.97513 | | 9.04 | | 73.85 | | 2 | |
|  | | 289-LEREWLR-295 |  | 1.00 | 2 | 1001.54344 | | -9.28 | | 51.10 | | 1 | |
|  | | 23-YHPTQPLHmK-32 | M31(Oxidation) | 0.98 | 2 | 1267.62065 | | -3.62 | | 53.45 | | 0 | |
|  | | 198-GqIIqVEAPWIK-209 | Q199(Deamidated); Q202(Deamidated) | 0.94 | 2 | 1383.74285 | | -6.55 | | 75.47 | | 0 | |
|  | | 264-LEPTLK-269 |  | 1.00 | 2 | 700.42626 | | 3.22 | | 46.61 | | 0 | |
|  | | 264-LEPTLKNAR-272 |  | **1.49** | 2 | 1041.60369 | | -1.43 | | 71.74 | | 1 | |
|  | | 233-TVTLGGIFQLGNWSGLNSVRDHNTIWK-259 |  | 1.38 | 3 | 3013.58017 | | 5.24 | | 107.58 | | 1 | |
|  | | 233-TVTLGGIFQLGNWSGLnSVR-252 | N249(Deamidated) | 1.21 | 3 | 2120.11631 | | 1.29 | | 116.56 | | 0 | |
|  | | 23-YHPTQPLHMKIYADR-37 |  | 0.95 | 2 | 1869.93230 | | -5.67 | | 95.89 | | 1 | |
| **Distal mucosa 40 kDa band:** | | | | | | | | | | | |  | |
|  | | **Sequence** | **Modifications** | **XCorr** | **Charge** | **MH+ [Da]** | | **ΔM [ppm]** | | **RT [min]** | | **Missed Cleavages** | |
|  | | 264-LEPTLK-269 |  | 1.00 | 2 | 700.42626 | | 3.22 | | 46.61 | | 0 | |
|  | | 264-LEPTLKNAR-272 |  | **1.49** | 2 | 1041.60369 | | -1.43 | | 71.74 | | 1 | |
|  | | 163-VESLEEVAR-171 |  | 1.16 | 2 | 1031.53874 | | 1.86 | | 48.04 | | 0 | |
|  | | 134-YHSVLQPLDLK-144 (A0A0G2JFX0 mouse isoform) |  | **2.83** | 2 | 1312.72490 | | -0.84 | | 67.39 | | 0 | |
|  | | 233-TVTLGGIFQLGnWSGLnSVRDHnTIWK-259 | N244(Deamidated); N249(Deamidated); N255(Deamidated) | **1.85** | 3 | 3016.49485 | | -7.16 | | 110.66 | | 1 | |
|  | | 115-KLTPSEmDLFPDYGYGWFnTSLLLEGK-141 | M121(Oxidation); N133(Deamidated) | **1.63** | 3 | 3138.51450 | | 4.06 | | 78.47 | | 1 | |
|  | | 86-mGLALISGYNLFRDEVPDPFWK-107 | M86(Oxidation) | 1.45 | 3 | 2584.28318 | | -2.87 | | 116.83 | | 1 | |
|  | | 23-YHPTqPLHmKIYADR-37 | Q27(Deamidated); M31(Oxidation) | 1.39 | 2 | 1886.92351 | | 0.89 | | 55.96 | | 1 | |
|  | | 172-GVDVIINcTGVWAGALQADASLqPGR-197 | C179(Carbamidomethyl); Q194(Deamidated) | 1.28 | 3 | 2669.34574 | | 3.90 | | 99.90 | | 0 | |
|  | | 233-TVTLGGIFqLGNWSGLnSVR-252 | Q241(Deamidated); N249(Deamidated) | 1.14 | 3 | 2121.10965 | | 5.69 | | 94.51 | | 0 | |
|  | | 108-nAVLGFRKLTPSEmDLFPDYGYGWFnTSLLLEGK-141 | N108(Deamidated); M121(Oxidation); N133(Deamidated) | 1.04 | 4 | 3896.92812 | | 4.82 | | 96.05 | | 2 | |
| **Medial epithelial layer 25 kDa band:** | | | |  |  |  | |  | |  | |  | |
|  | | **Sequence** | **Modifications** | **XCorr** | **Charge** | **MH+ [Da]** | | **ΔM [ppm]** | | **RT [min]** | | **Missed Cleavages** | |
|  | | 108-NAVLGFRKLTPSEmDLFPDYGYGWFnTSLLLEGK-141 | M121(Oxidation); N133(Deamidated) | **1.76** | 5 | 3895.94129 | | 4.10 | | 63.71 | | 2 | |
|  | | 108-nAVLGFR-114 | N108(Deamidated) | **1.68** | 2 | 777.42504 | | -0.48 | | 76.21 | | 0 | |
|  | | 151-LTERGVK-157 |  | **1.51** | 2 | 802.47777 | | -0.50 | | 17.23 | | 1 | |
| **Medial epithelial layer 20 kDa band:** | | | | | |  | |  | |  | |  | |
|  | | **Sequence** | **Modifications** | **XCorr** | **Charge** | **MH+ [Da]** | | **ΔM [ppm]** | | **RT [min]** | | **Missed Cleavages** | |
|  | | 264-LEPTLKNAR-272 |  | **1.43** | 2 | 1041.60625 | | 1.03 | | 50.03 | | 1 | |
| **Medial epithelial layer 15 kDa band:** | | | |  |  |  | |  | |  | |  | |
|  | | **Sequence** | **Modifications** | **XCorr** | **Charge** | **MH+ [Da]** | | **ΔM [ppm]** | | **RT [min]** | | **Missed Cleavages** | |
|  | | 86-MGLALISGYNLFRDEVPDPFWK-107 |  | **1.87** | 3 | 2568.31656 | | 8.13 | | 98.81 | | 1 | |

**Supplementary Table 6.** List of the peptides identified by mass spectrometry on mucosal fractions from wild-type and DAAO^-/-^ mice.

| **Sequence** | **Modifications** | **XCorr** | **Charge** | **MH+ [Da]** | **ΔM [ppm]** | **RT [min]** | **Missed Cleavages** |
| --- | --- | --- | --- | --- | --- | --- | --- |
| Wild-type proximal: | | | | | | | |
| 172-GVDVIIncTGVWAGALQADASLQPGR-197 | N178(Deamidated); C179(Carbamidomethyl) | 1.51 | 3 | 2669.36 | 7.88 | 111.00 | 0 |
| 163-VESLEEVAR-171 |  | 1.48 | 2 | 1031.53 | -6.66 | 71.98 | 0 |
| Wild-type medial: | | | | | | | |
| 198-GQIIqVEAPWIK-209 | Q202(Deamidated) | 1.80 | 3 | 1424.78 | 2.92 | 72.48 | 0 |
| 233-TVTLGGIFqLGNWSGLnSVR-252 | Q241(Deamidated); N249(Deamidated) | 1.50 | 3 | 2121.09 | -7.94 | 102.88 | 0 |
| 99-DEVPDPFWKnAVLGFRK-144 | N108(Deamidated) | 1.43 | 3 | 2019.05 | 3.37 | 70.38 | 2 |
| Wild-type distal: | | | | | | | |
| 162-KVESLEEVAR-171 |  | 1.72 | 3 | 1159.64 | 4.44 | 23.78 | 1 |
| 158-LIHRKVESLEEVAR-171 |  | 1.53 | 3 | 1678.94 | -9.18 | 54.12 | 2 |
| 172-GVDVIIncTGVWAGALQADASLQPGR-197 | N178(Deamidated); C179(Carbamidomethyl) | 1.44 | 3 | 2669.35 | 4.86 | 110.34 | 0 |
| 162-KVESLEEVARGVDVIINcTGVWAGALQADASLQPGR-197 | C179(Carbamidomethyl) | 1.42 | 5 | 3808.97 | 2.09 | 120.39 | 2 |
| DAAO^-/-^ mice proximal: | | | | | | | |
| not found |  |  |  |  |  |  |  |
| DAAO^-/-^ mice medial: | | | | | | | |
| not found |  |  |  |  |  |  |  |
| DAAO^-/-^ mice distal: | | | | | | | |
| not found |  |  |  |  |  |  |  |

**Supplementary Data 6. DAAO activity assay in mice gut samples**

With the aim to verify the presence of active DAAO in the intestinal fractions, two methods have been used: a) native PAGE: 50 µg of total proteins of each fraction were analysed by native electrophoresis (non-denaturating, 7.5% acrylamide resolving gel). Gels were stained with Coomassie blue or for DAAO activity based on the reduction of iodonitrotetrazolium salt, i.e by incubating at 37 °C the gel in 35 mM sodium pyrophosphate, pH 8.5, 0.2 mM FAD, 28 mM D-Ala, 0.18 mM iodonitrotetrazolium dissolved in ethanol [8]; b) Amplex UltraRed: DAAO activity was determined based on hydrogen peroxide formation. Mouse intestinal fractions (5, 10 and 20 µg of total proteins) were diluted in 50 mM Na_2_HPO_4_ pH 7.4. In a 96-wells plate, the samples were added to a solution of 56 µM Amplex UltraRed reagent (ThermoFisher Scientific), 0.15 U/mL horseradish peroxidase, 28 mM D-alanine, 7.5 mM sodium azide (to inhibit catalases) and 40 µM FAD in a total volume of 150 µL. The fluorescence intensity was recorded with a Tecan microplate using 535 nm excitation and 590 nm emission filters. Recombinant hDAAO and cerebellum extracts were used as positive controls while the assay specificity was evaluated omitting the substrate D-alanine or adding the inhibitor CBIO (1 mM) in the assay mixture.

Zymograms performed following native-PAGE on mice intestinal samples showed a faint positive band at low electrophoretic mobility, significantly different from the one observed for the reference recombinant hDAAO and from the signal observed using a mice cerebellum sample (Supplementary Fig. 5A, B). The altered migration in native-PAGE of hDAAO in crude samples could be ascribed to sample composition since also the recombinant hDAAO added to mucosal proximal mouse intestine samples did not migrate as the pure protein alone. Anyway, the activity signal was also apparent in samples from DAAO^-/-^ mice (Supplementary Fig. 5D, E) or when the substrate D-alanine was omitted from the developing mixture (Supplementary Fig. 5C, F), pointing to aspecific signals. As a further control, DAAO activity was assayed on small intestine samples using the sensitive Amplex UltraRed method that measures the H_2_O_2_ generated by the enzyme. As reported in Supplementary Fig. 6, the recorded DAAO activity signal is close to the detection limit for all tested samples and four order of magnitude lower than the value of recombinant hDAAO. Indeed, the experimental activity values were not modified by omitting the substrate D-alanine as well as by adding the inhibitor CBIO.

Altogether, we exclude the presence of DAAO activity in mice gut samples at a detectable level.

**
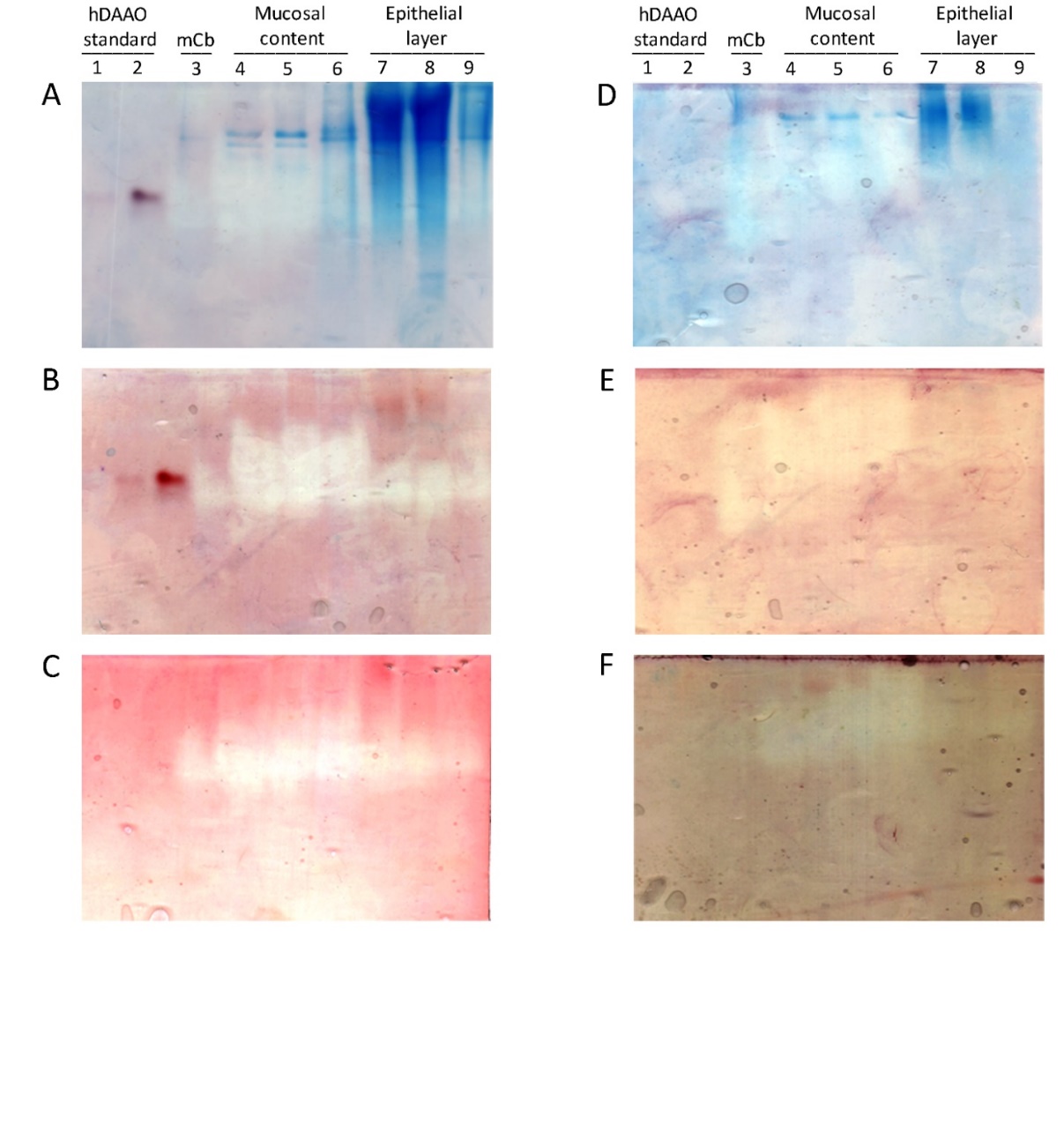
**

**Supplementary Fig. 5.** Coomassie blue (A, D) and activity staining (B, C, E, F) of gels from native-PAGE analysis of fractions (50 µg proteins) of small intestine from wild-type (A, B, C) and knock-out (D, E, F) mice (proximal, medial and distal mucosal content corresponding to lanes 4, 5 and 6; proximal, medial and distal epithelial layer, corresponding to lanes 7, 8 and 9). Recombinant hDAAO was used as reference (0.2 and 1 µg in lanes 1 and 2, respectively). Mouse cerebellum (lane 3) was also used as positive control. The specificity of the activity signal was verified removing the substrate from the developing solution (panels C and F).


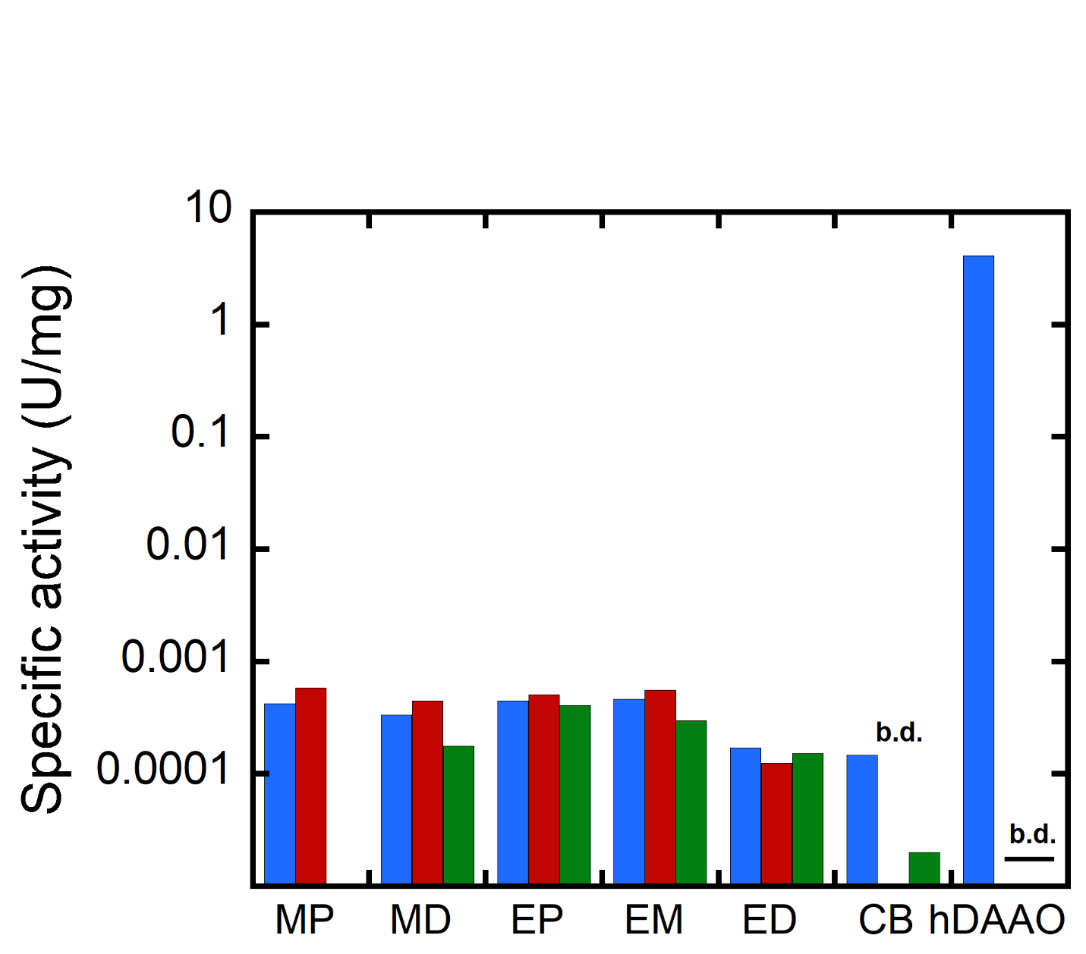


**Supplementary Fig. 6.** DAAO activity of samples from small intestine of wild-type mice (in blue). As control, the activity was assayed without the substrate D-Ala (in red) or adding 1 mM of the DAAO inhibitor CBIO (in green). MP = proximal mucosal content; MD = distal mucosal content; EP = proximal epithelial layer; EM = medial epithelial layer; ED = distal epithelial layer; CB = cerebellum; hDAAO = recombinant human DAAO; b.d. = below detection.

**Supplementary Data 7. CD analysis of CAMPs arising from DAAO**

Circular dichroism (CD) spectra in the far-UV region (190-250 nm) of 0.1 mg/mL DAAO-generated peptides were recorded in 10 mM sodium phosphate buffer pH 7.0 by using a Jasco J-815 spectropolarimeter (Jasco Co., Cremella, Italy) [9]. The effect of sodium dodecyl sulphate (SDS, 0.1-40 mM), 2,2,2-trifluoroethanol (TFE, 5-50% v/v) and lipopolysaccharides from *E. coli* (LPS, 0.1-1 mg/mL) was analyzed by titrating the peptides with increasing concentrations of these compounds. CD-spectra were analyzed using the Selcon3 method by DichroWeb [10] to estimate secondary structure content.


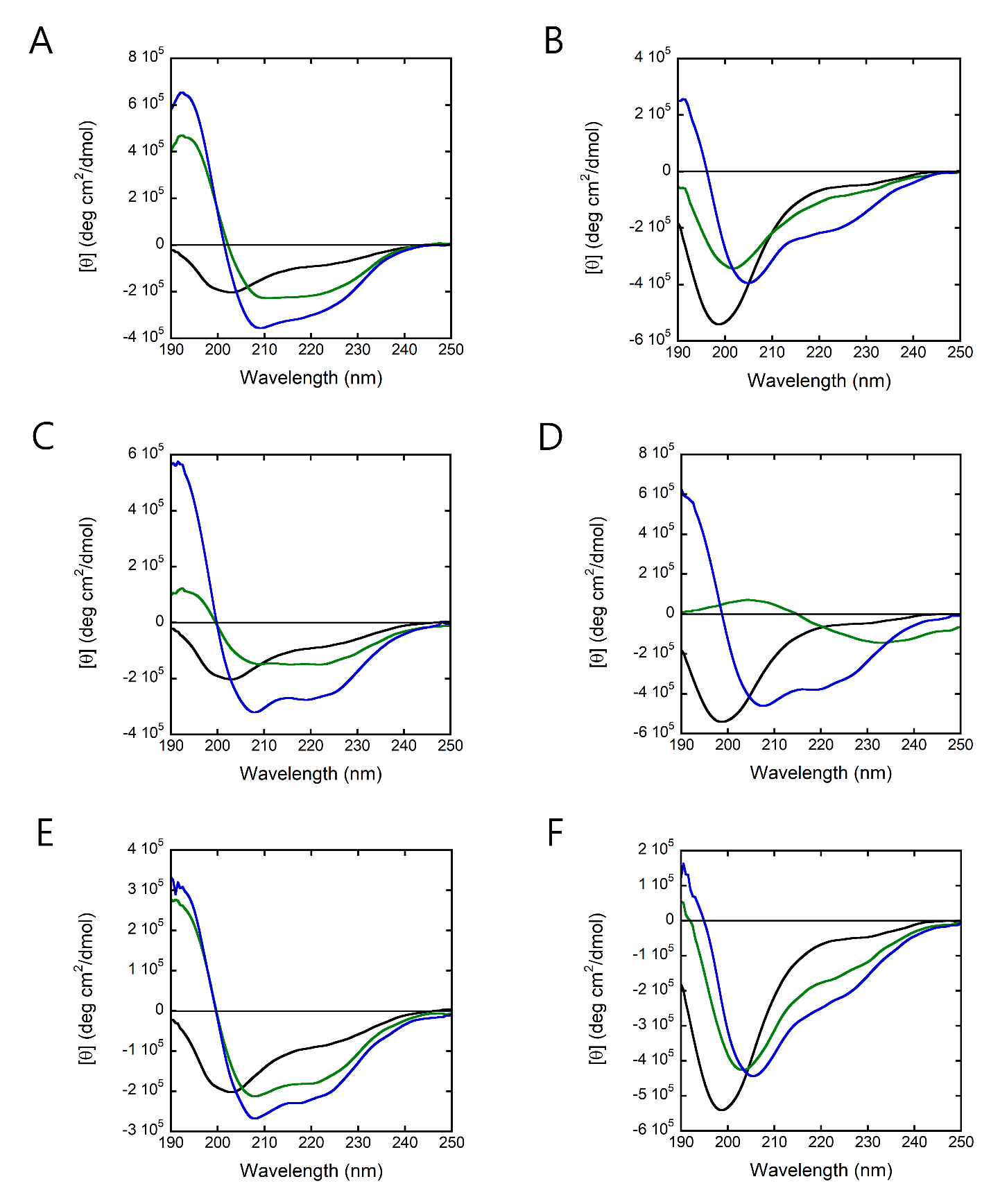


**Supplementary Fig. 7.** Comparison of far-UV CD spectra of DAAO antimicrobial peptides: GLT (A, C, E) and IWK (B, D, F) peptides (0.1 mg/mL, black) in the presence of: (A, B) 0.1 and 1 mM SDS (green and blue, respectively); (C, D) of 5 and 30% TFE (green and blue, respectively), (E, F) 0.1 and 0.25 mg/mL LPS (green and blue, respectively).

**Supplementary references**

1. Bendtsen JD, Nielsen H, von Heijne G, Brunak S (2004). Improved prediction of signal peptides: SignalP 3.0. J Mol Biol.; 340(4):783-795. doi:10.1016/j.jmb.2004.05.028

2. Bendtsen JD, Jensen LJ, Blom N, Von Heijne G, Brunak S (2004). Feature-based prediction of non-classical and leaderless protein secretion. Protein Eng Des Sel.; 17(4):349-356. doi:10.1093/protein/gzh037

3. Petersen TN, Brunak S, von Heijne G, Nielsen H (2011). SignalP 4.0: discriminating signal peptides from transmembrane regions. Nat Methods.; 8(10):785-786. doi:10.1038/nmeth.1701

4. Shen HB, Chou KC (2007). Signal-3L: A 3-layer approach for predicting signal peptides. Biochem Biophys Res Commun.; 363(2):297-303. doi:10.1016/j.bbrc.2007.08.140

5. Zhang YZ, Shen HB (2017). Signal-3L 2.0: A hierarchical mixture model for enhancing protein signal peptide prediction by incorporating residue-domain cross-level features. J Chem Inf Model.; 57(4):988-999. doi:10.1021/acs.jcim.6b00484

6. Molla G, Sacchi S, Bernasconi M, Pilone MS, Fukui K, Pollegioni L (2006). Characterization of human D-amino acid oxidase. FEBS Lett.; 580(9):2358-2364. doi:10.1016/j.febslet.2006.03.045

7. Romano D, Molla G, Pollegioni L, Marinelli F (2009). Optimization of human D-amino acid oxidase expression in Escherichia coli. Protein Expr Purif.; 68(1):72-78. doi:10.1016/j.pep.2009.05.013

8. Piubelli L, Molla G, Caldinelli L, Pilone MS, Pollegioni L (2003). Dissection of the structural determinants involved in formation of the dimeric form of D-amino acid oxidase from *Rhodotorula gracilis*: role of the size of the betaF5-betaF6 loop. Protein Eng.; 16(12):1063-1069. doi:10.1093/protein/gzg125

9. Caldinelli L, Molla G, Sacchi S, Pilone MS, Pollegioni L (2009). Relevance of weak flavin binding in human D-amino acid oxidase. Protein Sci.; 18(4):801-810. doi:10.1002/pro.86

10. Whitmore L, Wallace BA (2008). Protein secondary structure analyses from circular dichroism spectroscopy: methods and reference databases. Biopolymers.; 89(5):392-400. doi:10.1002/bip.20853
